# Activation of the TGFβ pathway impairs endothelial to haematopoietic transition

**DOI:** 10.1038/srep21518

**Published:** 2016-02-19

**Authors:** Özge Vargel, Yang Zhang, Kinga Kosim, Kerstin Ganter, Sophia Foehr, Yannicka Mardenborough, Maya Shvartsman, Anton J. Enright, Jeroen Krijgsveld, Christophe Lancrin

**Affiliations:** 1 European Molecular Biology Laboratory, Mouse Biology Unit, Via Ercole Ramarini 32, 00015 Monterotondo, Italy; 2 European Molecular Biology Laboratory, Genome Biology Unit, Meyerhofstraße 1, 69117 Heidelberg, Germany; 3 European Molecular Biology Laboratory, European Bioinformatics Institute, Wellcome Genome Campus, Hinxton, Cambridge, CB10 1SD, United Kingdom

## Abstract

The endothelial to haematopoietic transition (EHT) is a key developmental process where a drastic change of endothelial cell morphology leads to the formation of blood stem and progenitor cells during embryogenesis. As TGFβ signalling triggers a similar event during embryonic development called epithelial to mesenchymal transition (EMT), we hypothesised that TGFβ activity could play a similar role in EHT as well. We used the mouse embryonic stem cell differentiation system for *in vitro* recapitulation of EHT and performed gain and loss of function analyses of the TGFβ pathway. Quantitative proteomics analysis showed that TGFβ treatment during EHT increased the secretion of several proteins linked to the vascular lineage. Live cell imaging showed that TGFβ blocked the formation of round blood cells. Using gene expression profiling we demonstrated that the TGFβ signalling activation decreased haematopoietic genes expression and increased the transcription of endothelial and extracellular matrix genes as well as EMT markers. Finally we found that the expression of the transcription factor Sox17 was up-regulated upon TGFβ signalling activation and showed that its overexpression was enough to block blood cell formation. In conclusion we showed that triggering the TGFβ pathway does not enhance EHT as we hypothesised but instead impairs it.

Haematopoietic stem and progenitor cells (HSPC) arise during embryonic life through a process called endothelial to haematopoietic transition (EHT)^1^,^2^,^3^,^4^,^5^. This is an evolutionary conserved embryonic process, which takes place in mammals and lower vertebrates such as fishes and frogs^6^. The EHT requires the loss of endothelial features and acquisition of haematopoietic ones. Loss of cell-cell adhesion between endothelial cells is required to enable the release of the HSPC in the blood stream. Signalling pathways responsible for this process are not well characterized.

Epithelial cells can convert to another cell type called mesenchymal cell by a process called epithelial to mesenchymal transition (EMT). It was first observed in the primitive streak of chick embryos^7^. It was later demonstrated to be an important process in the formation of metastasis in cancers occurring in epithelial tissues^8^. EMT leads to the loss of cell-cell interaction and organisation of an epithelial cell layer. It involves major changes in adhesion properties, morphology and mobility. Interestingly, the endothelial cells can also undergo a specific form of EMT, the endothelial to mesenchymal transition (EndMT) by which endothelial cells become mesenchymal, acquiring morphological features compatible with cell mobility and migration^9^. It takes place during embryonic development to enable the formation of endocardial cushions^10^ and can be involved in diseases such as the fibrodysplasia ossificans progressiva (FOP)^11^ and cerebral cavernous malformations^12^.

The transforming growth factor beta (TGFβ) signalling pathway has been shown to induce EMT and EndMT^8^,^9^. The pathway consists of three main players: TGFβ ligands, cell membrane bound receptors and intracellular effector molecules^13^. The signalling is initiated upon binding of a ligand on a homodimer of TGFβ receptor type II (TGFBR2). This binding recruits a TGFβ receptor type I homodimer such as TGFBR1 (ALK5) to form all together a hetero-tetrameric complex while TGFBR2 phosphorylates the type I receptors. Phosphorylated type I receptors become active and then phosphorylate and activate a group of receptor regulated SMAD (R-SMAD) proteins (SMAD2 and 3 for TGFBR1). The phosphorylated R-SMADs later form heterodimers with the common mediator SMAD proteins (SMAD4) and localize into the nucleus where they activate transcription of target genes. Another type of SMAD proteins, inhibitory SMAD (SMAD6 and 7), can block the phosphorylation of R-SMADs, which cuts off the down-stream relay of the signal^13^. TGFBR1 and SMAD3 are key players in the induction of EMT by activating the expression of the transcription factor SNAIL^14^.

Embryonic stem cell (ESC) differentiation model has been used extensively to study embryonic haematopoiesis and has been instrumental in our understanding of key events happening during formation of blood cells^15^. Haematopoietic progenitor cells (HPC) are also formed through the process of EHT in the ESC differentiation model^16^,^17^. We have used this system to test whether or not activation of TGFβ signalling enhances EHT like it does for EMT and EndMT. We used a wide range of methods from quantitative RT-PCR, quantitative proteomics analysis of the secretome, ESC differentiation, flow cytometry, live cell imaging and mRNA microarrays to study the impact of loss and gain of function of the TGFβ signalling on the formation of HPCs. Surprisingly, unlike the promoting effect of TGFβ in EMT and EndMT, we found that the TGFβ signalling inhibits EHT.

## Results

### TGFβ pathway related genes are expressed during EHT

**Figure 1:**
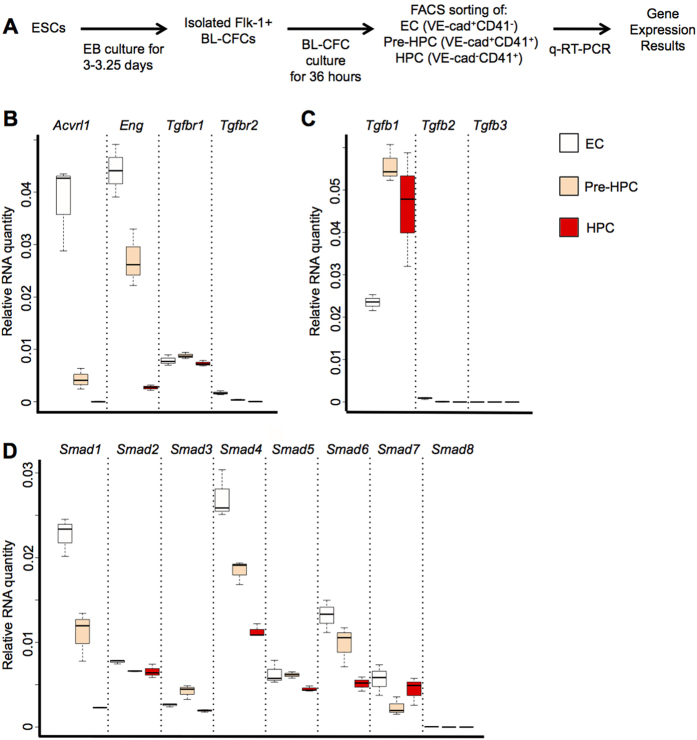
TGFβ pathway related genes are expressed during *in vitro* EHT. (**A**) Experimental workflow used to generate the indicated 3 populations from ESC. (**B**) Expression of TGFβ receptor coding genes in EC, Pre-HPC and HPC populations. (**C**) Expression of TGFβ ligand coding genes in EC, Pre-HPC and HPC populations. (**D**) Expression of SMAD coding genes in EC, Pre-HPC and HPC populations. The box plots were generated from 3 independent experiments. For each plot, the top and bottom box edges correspond to the first and third quartiles. The black line inside the box represents the median. The top and bottom whisker lines mark the maximum and minimum values of the data set, respectively. The corresponding p-values were calculated with Student’s t-test (Supplementary Table S1).

Using the embryonic stem cell differentiation system it is possible to recapitulate the first events of blood cell development taking place in the mouse embryo^18^. After isolating mesodermal Flk1^+^ containing Blast-Colony-Forming Cells (BL-CFC), common precursor of blood and endothelial cells^19^,^20^, from day 3–3.25 embryoid body (EB) differentiation and putting them in culture in presence of VEGF and IL6, we can follow the formation of blood cells from endothelial cells (EC). They differentiate into haematopoietic progenitor cells (HPC) via the formation of an intermediary stage called pre-haematopoietic progenitor cells (Pre-HPC). We isolated each population based on the expression of the endothelial marker VE-Cadherin (VE-Cad)^21^ and the haematopoietic marker CD41^17^ by flow cytometry after 1.5 days of BL-CFC culture (Fig. 1A and Supplementary Fig. S1A): VE-Cad^+^CD41^−^ (EC) expressing high level of endothelial genes (*Cdh5*, *Kdr* and *Pecam1*) but low level of blood genes (*Runx1*, *Myb* and *Gata1*), VE-Cad^+^CD41^+^ (Pre-HPC) co-expressing endothelial and haematopoietic genes and VE-Cad^−^CD41^+^ (HPC) expressing only haematopoietic genes (Supplementary Fig. S1B and S1C). To find out if any of these populations could respond to TGFβ signalling, we performed quantitative RT-PCR (q-RT-PCR) to detect 15 genes coding for proteins involved in the TGFβ signalling pathway: 4 receptors, 3 ligands and 8 SMADs (Fig. 1 and Supplementary Table S1). All these genes were transcribed in the EC population although *Tgfb3* and *Smad8* were expressed at a low level. The Pre-HPC population expressed many of these genes although there was a clear drop in expression levels for *Acvrl1*, *Tgfbr2* and *Tgfb2* that were even lower in HPC. All populations expressed the *Smad* genes and *Tgfbr1;* however *Tgfbr2* and *Acvrl1* were only detected in EC and Pre-HPC suggesting that these 2 populations were the most likely to respond to TGFβ signalling. This gene expression analysis indicated that the TGFβ signalling could play a role in EHT.

### TGFβ2 treatment during BL-CFC culture increases the secretion of proteins linked with vascular development, extracellular matrix and cell mobility

Between day 1 and day 2 of BL-CFC culture, there is a clear increase of CD41^+^ blood precursors suggesting that endothelial to haematopoietic transition occurs during that time^17^. To get an insight into proteins involved in cellular communication, adhesion and migration occurring during EHT, we performed a secretome analysis of BL-CFC cells to detect the proteins secreted upon TGFβ pathway activation compared to control conditions. If the TGFβ signalling promotes EHT, we would expect the secretion of proteins linked to haematopoiesis. To test this hypothesis, we performed stable isotope labelling with amino acids in cell culture (SILAC) in combination with labelling with azidohomoalanine (AHA). AHA, an artificial amino-acid analogue of methionine permits the capture of newly synthesized proteins through click chemistry followed by mass spectrometry for protein identification^22^.

**Figure 2:**
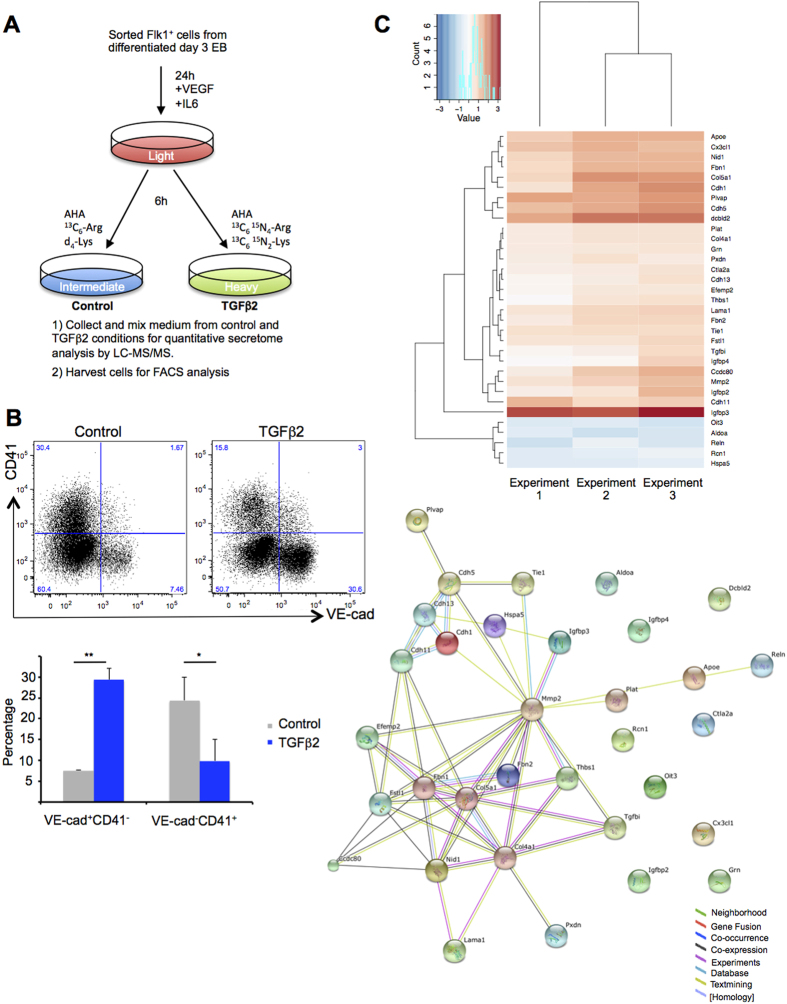
TGFβ treatment during BL-CFC culture favours the vascular lineage over the haematopoietic one. (**A**) Experimental workflow of the quantitative secretome analysis by LC-MS/MS. (**B**) Flow cytometry analysis of VE-Cad and CD41 expression after 6 hours of culture following the addition of TGFβ2 compared to control condition. Upper panel shows one representative example of flow cytometry analysis while the bottom one shows a bar graph representing the average frequency of the EC (VE-Cad^+^CD41^−^) and HPC (VE-Cad^−^CD41^+^) populations from 3 independent experiments. The p-values were calculated with Student’s t-test (2 tails, type 3). EC population: **Control versus TGFβ2 p-value = 0.004 (n = 3); HPC population: *Control versus TGFβ2 p-value = 0.03 (n = 3). (**C**) Heatmap representing Log_2_ fold change (Log_2_ FC) of protein expression between TGFβ2 treated and non-treated samples for all the proteins detected in the 3 biological replicates. Each column represents one independent secretome experiment. (**D**) STRING representation of a network involving the proteins detected in C. Nine proteins out of 33 were not part of the network. Different line colours represent the types of evidence for the association between proteins.

After 1 day of BL-CFC culture, cells were treated for 6 hours with TGFβ2^11^ or with the vehicle as a negative control in presence of AHA, ‘intermediate’ or ‘heavy’ isotopes of lysine and arginine. Supernatants from the 2 conditions were harvested and pooled; AHA-containing proteins were isolated with click chemistry and subjected to liquid chromatography tandem mass spectrometry (LC-MS/MS) (Fig. 2A). In the meantime the cells were harvested for flow cytometry analysis and examined for the expression of CD41 and VE-Cad markers. Surprisingly we noticed that the 6 hours TGFβ2 treatment increased the frequency of EC (VE-Cad^+^CD41^−^) by 4-fold compared to the non-treated condition whereas the frequency of HPC (VE-Cad^−^CD41^+^) was reduced by roughly 2-fold (Fig. 2B).

In the three biological replicates we could identify between 133 and 268 proteins newly synthesized during the 6 hours pulse (Supplementary Table S2). The number of proteins with secreted evidence varied between 80 and 100 (Supplementary Fig. S2A). Pairwise comparisons between these samples showed correlation values between 0.78 and 0.92 (Supplementary Fig. S2B). Fifty-six proteins were differentially expressed in all 3 replicates including 33 with secreted evidence. According to the STRING analysis tool^23^, the majority of these proteins had interactions between them (Fig. 2C). Interestingly, 85% of these secreted proteins had an increased expression after TGFβ2 treatment (Fig. 2B and Supplementary Table S3). Among them DCBLD2 is important for angiogenesis^24^; CX3CL1 (also known as Fractalkine) is a chemokine produced by endothelial cells^25^; COL5A1 is crucial for cardiovascular development^26^; PLVAP helps to maintain vascular permeability of endothelial cells^27^ and MMP2 is a target of TGFβ signalling and an important player in EMT^28^,^29^. Gene ontology (GO) analysis indicated enrichment in proteins involved in cell migration, extracellular matrix organization and vasculature development (Supplementary Fig. S3). Although Csf1^30^, a cytokine supporting macrophage growth, was detected, its expression was not up-regulated after TGFβ2 treatment (Supplementary Table S2).

Overall, this first series of experiments suggests that the TGFβ pathway does not enhance haematopoietic development. On the contrary, only 6 hours of TGFβ treatment was sufficient to increase clearly the frequency of the endothelial cells and the quantity of secreted proteins related with vascular development, extracellular matrix organisation and cell migration.

### TGFβ pathway activation blocks EHT

**Figure 3:**
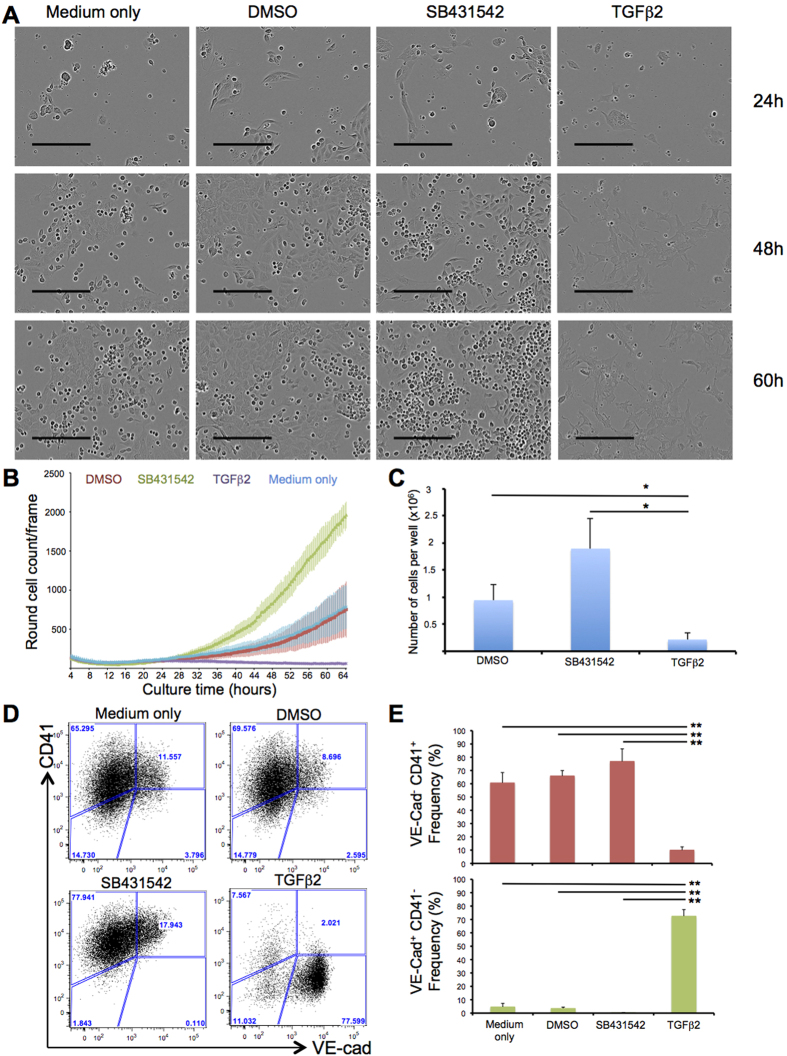
TGFβ treatment blocks blood cell formation, reduces the frequency of haematopoietic cells and increases the frequency of endothelial cells. (**A**) Images of the haemogenic endothelium (HE) culture taken after 24, 48 and 60 hours in different conditions. The scale bar corresponds to 200 μm. (**B**) Quantification of the number of round cells generated during time-lapse imaging in the 4 indicated conditions. Each value represents the mean of the number of round cells for 9 areas of the same well. Error bars represent standard deviation. (**C**) Bar graphs representing the average cell number of the 3 indicated conditions from 3 independent experiments. Error bars correspond to standard deviation. The p-values were calculated with Student’s t-test (2 tails, type 3). *DMSO versus TGFβ2 p-value = 0.035 (n = 3); *SB431542 versus TGFβ2 p-value = 0.03 (n = 3). (**D**) Flow cytometry analysis of VE-Cad and CD41 expression after 3 days of culture following the addition of TGFβ2 compared to control condition for one representative experiment. (**E**) Bar graphs representing the average frequency of the 2 indicated populations from 3 independent experiments. Error bars correspond to standard deviation. The p-values were calculated with Student’s t-test (2 tails, type 3). VE-Cad^−^ CD41^+^ population p-values: **Medium only versus TGFβ2 p-value = 0.004 (n = 3); **DMSO versus TGFβ2 p-value = 0.004 (n = 3); **SB431542 versus TGFβ2 p-value = 0.0001 (n = 3). VE-Cad^+^ CD41^−^ population p-values: **Medium only versus TGFβ2 p-value = 0.00018 (n = 3); **DMSO versus TGFβ2 p-value = 0.0011 (n = 3); **SB431542 versus TGFβ2 p-value = 0.0015 (n = 3).

Our previous experiments suggested an inhibitory effect of TGFβ on EHT. We sought to verify this by using a different assay. For this new analysis we focused on Pre-HPC (VE-Cad^+^CD41^+^). When these cells were isolated and put in culture most of them still had an endothelial cell morphology after 24 hours (Fig. 3A). One of the landmarks of blood cell formation is the change in morphology from flat and adherent to round and floating (Fig. 3A). We decided to choose this step of EHT as an ideal read-out to analyse the impact of TGFβ activity. In order to activate the TGFβ signalling pathway, we added TGFβ2 into the culture medium. Since TGFBR1 activation has been shown to trigger EMT^14^ we chose to use SB431542^31^ to inhibit the pathway. As a control, we used normal culture medium with the same volume of vehicle (DMSO for SB431542) or without anything (Supplementary Fig. S4).

The cells were re-plated in haemogenic endothelium mix^17^ with either nothing, DMSO, SB431542 or TGFβ2 for 3 days. The time-lapse images were taken for 3 days every 15 minutes. The number of round cells during the 3 days of culture was quantified by analysing the images with the CellProfiler software. The DMSO control was very similar to the control with nothing added (Fig. 3, Supplementary Videos S1&S2). However there was a profound difference between the cells treated with SB431542 or TGFβ2. In presence of TGFβ2, we could clearly see a drastic decrease of round cells’ number (Fig. 3 and Supplementary Video S3) while the treatment with SB431542 led to an increase in the number of round cells (Fig. 3 and Supplementary Video S4). In accordance to the microscopy data, the number of cells after TGFβ treatment decreased while the number of cells increased after SB431542 addition (Fig. 3C). At the end of the culture, the cells were examined for VE-Cad and CD41 expression by flow cytometry analysis. The cells were expected to be mostly HPC (VE-Cad^−^CD41^+^) with very few cells remaining Pre-HPC (VE-Cad^+^CD41^+^). This was what we observed for the conditions Medium only and DMSO (Fig. 3D,E). In the SB431542 condition, we noticed a slight increase of HPC frequency compared to controls. On the other hand we observed a very low frequency of HPC (around 7 times less than in the other 3 conditions) in the TGFβ2 treated samples suggesting that TGFβ2 inhibits EHT. Moreover the majority of cells at the end of the culture have lost their Pre-HPC phenotype and adopted an EC phenotype (VE-Cad^+^ CD41^−^) instead (Fig. 3D,E). This FACS analysis was both consistent with the microscopy analysis and secretome analysis of Fig. 2.

To confirm the effect of SB431542 on EHT, we compared its activity to the over-expression of Smad7, another inhibitor of TGFBR1^32^,^33^. We made an inducible cell line where GFP-Smad7 could be induced upon addition of doxycycline (dox) (Supplementary Fig. S5A & S5B). We first showed that SB431542 treatment and Smad7 overexpression for 24 hours both decreased the frequency of endothelial cells by 2-fold in the BL-CFC assay (Supplementary Fig. S5C). This phenotype is in accordance with the increase of endothelial cells’ frequency observed after TGFβ2 treatment in Fig. 2B.

When we tested the over-expression of Smad7 at the pre-HPC stage, we observed only a small increase in round cells’ number in presence of dox compared to the no dox control (Supplementary Fig. S5D). This suggests that the overexpression of Smad7 could not fully recapitulate SB431542 activity.

In conclusion, microscopy and flow cytometry data reinforced the notion that the TGFβ signalling does not enhance EHT. On the contrary, TGFβ activation resulted in almost no round cells but more flat and scattered VE-Cad^+^CD41^−^ cells (Fig. 3 and Supplementary Video S3).

### Gene expression profiling after activation and inhibition of the TGFβ signalling pathway confirms the inhibitory role of TGFβ in EHT

**Figure 4:**
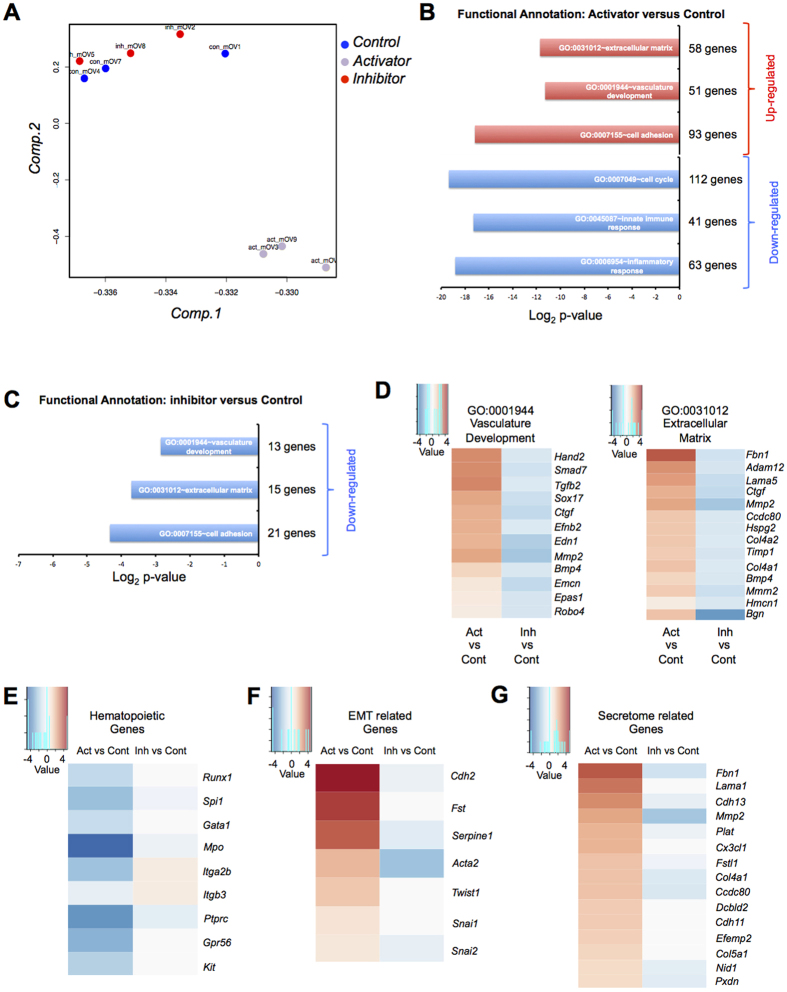
mRNA microarray analysis reveals the impact of TGFβ signalling activation or inhibition at the transcriptional level. (**A**) Principal component analysis of the samples. (**B**) Functional annotation for the “Activator versus Control” contrast. The log_2_ p-value was plotted for each GO term. The number of genes per group is also indicated. (**C**) Functional annotation for the “Inhibitor versus Control” contrast. The log_2_ p-value was plotted for each GO term. The number of genes per group is also indicated. (**D**) Heatmap of Log_2_ FC of genes from the vasculature development (left) and extracellular matrix (right) GO terms. Log_2_ FC values obtained from Activator versus Control (ActvsCont) contrast were compared to the ones from Inhibitor versus Control (InhvsCont) contrast. (**E**) Heatmap of Log_2_ FC of haematopoietic genes. (**F**) Heatmap of Log_2_ FC of EMT genes. (**G**) Heatmap of Log_2_ FC of candidate genes corresponding to detected proteins in Fig. 2.

We performed mRNA microarray analysis of the 3 sets of cells: treated with DMSO (control), with SB431542 (inhibitor) and with TGFβ2 (activator) (Fig. 4A, Supplementary Fig. S4 and Supplementary Table S4). Principal Component Analysis (PCA) showed clearly that the activator samples were very different than the other two. Control and inhibitor samples were clustering together indicating similar expression profile (Fig. 4A). Hierarchical clustering analysis with the top 250 differentially expressed genes confirmed the results of the PCA (Supplementary Fig. S6). This gene expression analysis was consistent with the flow cytometry data described previously where the biggest difference was between the cells treated with TGFβ2 and the other conditions (Fig. 3).

Differential gene expression analysis between control and activator samples generated a list of over 2400 genes whose expression was changed by at least 2-fold between the 2 conditions (Supplementary Table S4). DAVID Gene ontology analysis^34^ showed that the activator treatment up-regulated the expression of genes linked to extracellular matrix, vasculature development and cell adhesion (Fig. 4B and Supplementary Table S5). These GO terms were the same as the ones we found for the secreted proteins in Supplementary Fig. S3. On the other hand, the down-regulated genes were associated to the GO terms cell cycle, immune response and inflammatory response (Fig. 4B). This strongly supported the notion of the inhibition of blood cell development in presence of TGFβ2. Moreover the reduction of cell cycle related genes could be associated to the inhibitory effect on the cell cycle of the TGFBR1 activation^35^. This is consistent with the decreased cell number observed after TGFβ2 treatment (Fig. 3C).

The list of genes differentially expressed between control and inhibitor samples was much smaller. Upon TGFβ pathway inhibition there were only 62 up-regulated (>2 fold) genes while 168 genes were down-regulated more than 2 fold compared to the control (Supplementary Table S4). This was consistent with the PCA and hierarchical clustering showing high expression similarities between them. Nonetheless, there were clear differences in the list of down-regulated genes in response to SB431542 treatment. These genes were related to vasculature development, extracellular matrix and cell adhesion GO terms (Fig. 4C). Most of the down-regulated genes in presence of inhibitor were up-regulated in presence of TGFβ2 (Fig. 4D).

We demonstrated in previous experiments a dramatic reduction of blood cell generation in presence of TGFβ2. This was supported by a clear down-regulation of many important haematopoietic genes such as *Runx1*, a crucial regulator of EHT^4^,^17^, *Spi1*, a key transcription factor for the development of myeloid and lymphoid lineages^36^ and *Gata1*, an essential gene for the formation of red blood cells^37^ (Fig. 4E).

The previous microscopy analysis showed that activation of the TGFβ signalling led to a mesenchymal morphology compatible with an EMT process. We found a set of up-regulated genes related to the GO terms of mesenchymal cell differentiation and mesenchyme development (Supplementary Table S5). To confirm this observation, we looked for EMT markers, *Cdh2, Fst, Serpine1, Acta2, Twist1, Snai1 and Snai2*^8^. They were all up-regulated after TGFβ signalling activation compared to control (Fig. 4F). In contrast, the difference between inhibitor and control samples were very small reflecting the already low level of expression of these genes in normal conditions.

In the proteome analysis, we identified 33 proteins whose quantity in the extracellular space was changed upon TGFβ2 treatment. The genes coding for these proteins belonged to the GO terms vasculature development, extracellular matrix and cell adhesion (Supplementary Fig. S3). We therefore looked for the genes coding for these proteins in the microarray data and found that 15 of them were clearly more expressed in presence of TGFβ2, which is consistent with the change of protein quantity we found in our secretome experiment (Fig. 2C). Only 2 of them (*Fbn1* and *Mmp2*) were down-regulated after SB431542 treatment (Fig. 4G).

**Figure 5:**
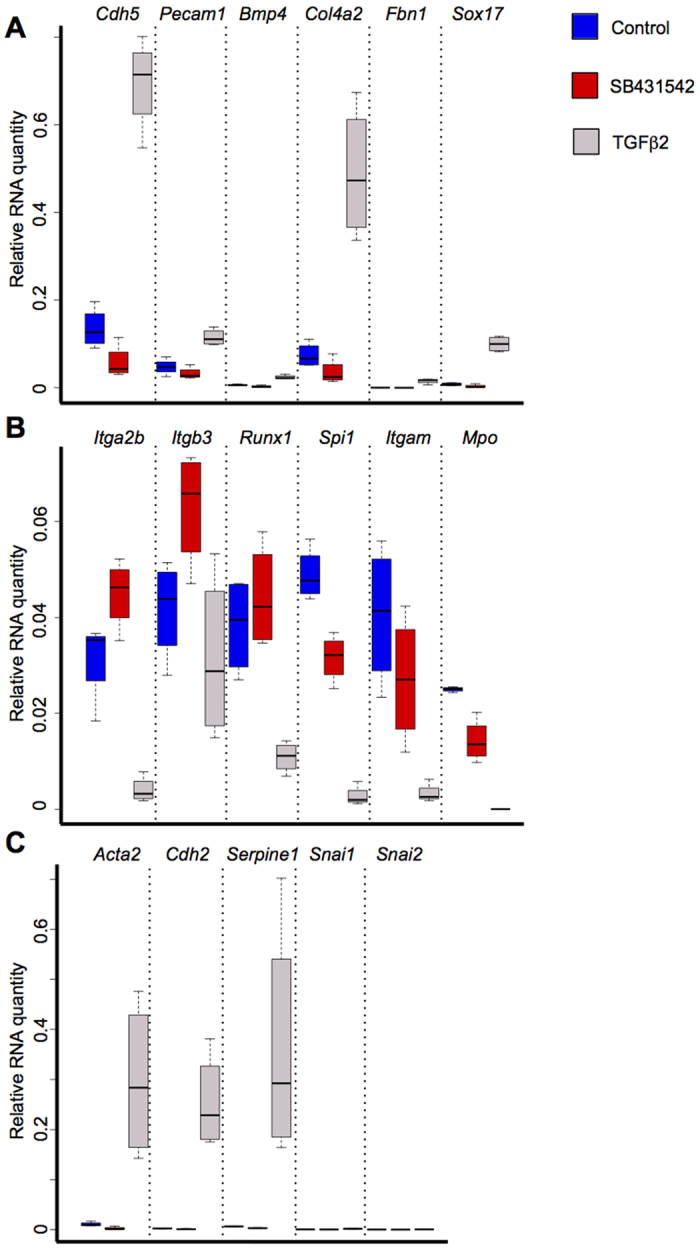
TGFβ signalling activation decreases haematopoietic gene expression and increases EMT and vascular genes transcription. (**A**) Box plots comparing the expression of vascular specific genes between the control, SB431542 and TGFβ2 conditions. (**B**) Box plots comparing the expression of haematopoietic genes between the 3 conditions. (**C**) Box plots comparing the expression of EMT specific genes between the 3 conditions. The box plots were generated from 4 independent experiments. For each plot, the top and bottom box edges correspond to the first and third quartiles. The black line inside the box represents the median. The top and bottom whisker lines mark the maximum and minimum values of the data set, respectively. The corresponding p-values were calculated with Student’s t-test (Supplementary Table S7).

To confirm the microarrays results, we performed q-RT-PCR for a selection of 17 genes on cDNA from Control, SB431542 and TGFβ2 conditions. We examined the expression of 6 genes related to the vascular lineages: *Cdh5, Pecam1, Bmp4, Col4a2, Fbn1 and Sox17*. We found that they were all up-regulated upon TGFβ2 treatment confirming the microarray analysis (Fig. 5A and Supplementary Table S7).

We studied the expression of 6 haematopoietic genes, *Itga2b, Itgb3, Runx1, Spi1, Itgam* and *Mpo*. As expected most of these genes were down-regulated upon TGFβ2 treatment (Fig. 5B). Only *Itgb3* expression was not significantly different between DMSO and SB431542 conditions (Supplementary Table S7).

Finally, we tested the expression of EMT genes *Acta2, Cdh2, Serpine1, Snai1 and Snai2*. *Acta2, Cdh2 and Serpine1* were strongly up-regulated after TGFβ signalling activation whereas a very modest increase of *Snai1* (p-value = 0.01) and *Snai2* (p-value = 0.046) expression was observed (Fig. 5C and Supplementary Table S7). We noticed a small but significant decrease for *Acta2* and *Serpine1* expression after SB431542 treatment (Fig. 5C and Supplementary Table S7).

In conclusion, our gene expression analysis confirmed the inhibitory effect of TGFβ on the EHT. TGFβ signalling activation reduced hematopoietic gene expression and increased the expression of endothelial genes and EMT markers.

### TGFβ pathway related genes are expressed during EHT in the Aorta Gonad Mesonephros region

**Figure 6:**
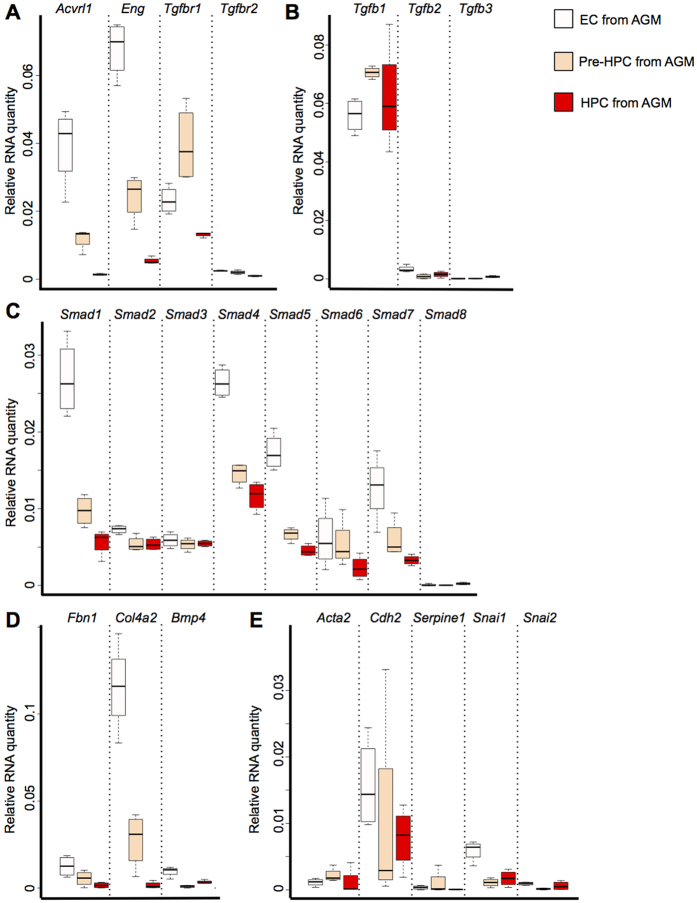
TGFβ pathway related genes are expressed during EHT in the Aorta Gonad Mesonephros (AGM) region. (**A**) Box plots comparing the expression of TGFβ receptors between the EC, Pre-HPC and HPC populations isolated from E11 AGM region. (**B**) Box plots comparing the expression of TGFβ ligands coding genes between the EC, Pre-HPC and HPC populations. (**C**) Box plots comparing the expression of SMAD coding genes in EC, Pre-HPC and HPC populations. (**D**) Box plots comparing the expression of vascular and extracellular matrix genes in EC, Pre-HPC and HPC populations. (**E**) Box plots comparing the expression of EMT specific genes in EC, Pre-HPC and HPC populations. The box plots were generated from 4 independent experiments. For each plot, the top and bottom box edges correspond to the first and third quartiles. The black line inside the box represents the median. The top and bottom whisker lines mark the maximum and minimum values of the data set, respectively. The corresponding p-values were calculated with Student’s t-test (Supplementary Table S8).

Next we decided to study the role of TGFβ pathway in the EHT taking place in the Aorta Gonad Mesonephros (AGM) region, one of the main sites of blood cell formation in the mouse embryo^6^. We sorted the EC (VE-Cad^+^CD41^−^), Pre-HPC (VE-Cad^+^CD41^+^) and HPC (VE-Cad^−^CD41^+^Kit^+^) populations from E11 AGM. As for the *in vitro* EHT populations, EC express high level of endothelial genes (*Cdh5*, *Kdr* and *Pecam1*) but low level of blood genes (*Runx1*, *Myb* and *Gata1*), Pre-HPC co-express endothelial and haematopoietic genes and HPC express only haematopoietic genes (Supplementary Fig. S7). We performed PCR for the TGFβ pathway related genes (Fig. 6 and Supplementary Table S8) and found an expression pattern very similar to the ones observed for the *in vitro* generated populations (Fig. 1). The EC population expressed all the tested TGFβ pathway genes while they were gradually down-regulated during EHT at the exception of *Tgfb1*, *Smad2* and *Smad3*. As for the *in vitro* generated EC and Pre-HPC populations, their *in vivo* counterparts were the most likely to respond to TGFβ signalling. *Fbn1*, *Col4a2* and *Bmp4*, vascular and extracellular matrix genes identified in the gene expression analysis (Figs 4 and 5), were all up-regulated upon TGFβ activation and they had a higher level of transcription in the EC population compared to the other groups (Fig. 6D and Supplementary Table S8). We observed the same expression pattern with the *in vitro* generated populations (Supplementary Fig. S8)

TGFβ triggers the expression of markers characteristic of EMT. We checked whether or not these genes could be associated with the progression of blood cell formation *in vivo*. We examined the expression of the EMT related genes, *Acta2*, *Cdh2*, *Serpine1*, *Snai1* and *Snai2*. Interestingly most of them were expressed at a low level and had a similar transcriptional level in all 3 populations. Only *Cdh2* and *Snai1* were expressed higher in the EC population compared to the other 2 (Fig. 6E and Supplementary Table S8). It showed clearly that the expression of these markers was not related to blood cell formation. Moreover, in the *in vitro* generated populations, *Acta2, Cdh2, Serpine1* and *Snai1* were all expressed higher in the EC group than in the Pre-HPC and HPC populations (Supplementary Fig. S8 and Supplementary Table S1). In conclusion we showed that EMT markers are not a good indicator of the EHT process and that genes whose expression was up-regulated by the TGFβ treatment are more expressed in the endothelial lineage.

### Overexpression of Sox17 during EHT partially recapitulates the effects of TGFβ signalling activation

Using several assays we demonstrated that TGFβ signalling activation inhibits the formation of blood cells. To know how TGFβ mediates this inhibition, we looked for candidate genes. We found that following TGFβ2 treatment, the expression of the gene *Sox17* was increased. This gene is crucial to acquire and maintain arterial identity^38^. We hypothesised that its higher expression after TGFβ treatment could maintain the expression of endothelial genes and therefore block EHT.

**Figure 7:**
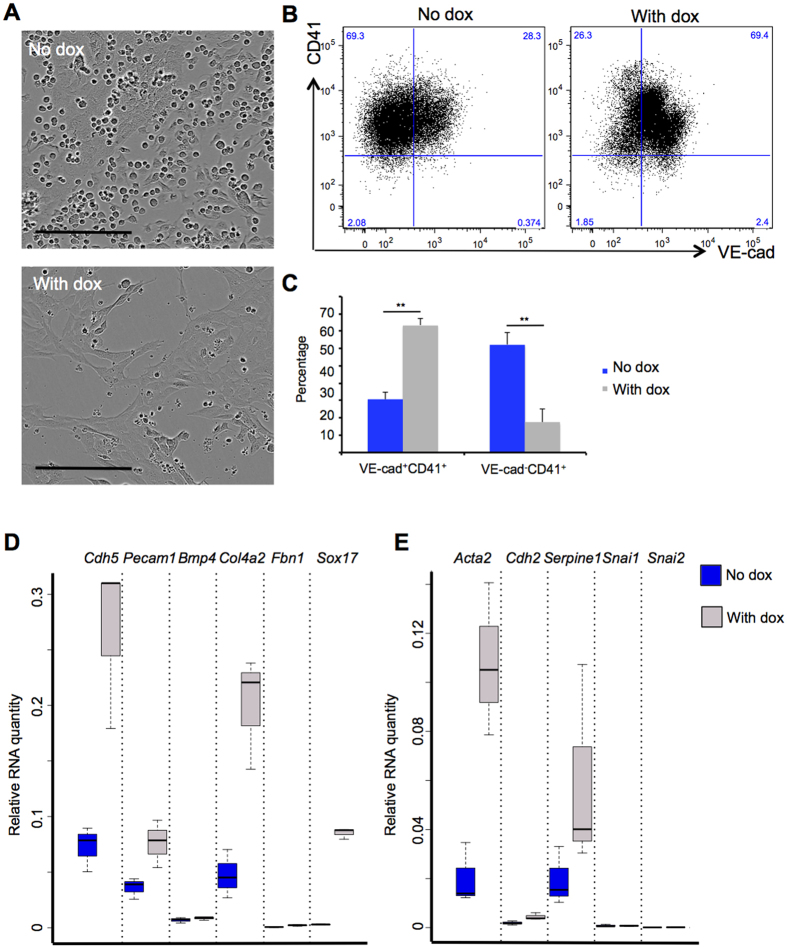
Overexpression of Sox17 during EHT partially recapitulates the effect of TGFβ signalling activation. (**A**) Images of the haemogenic endothelium (HE) culture taken after 48 h in absence or presence of doxycycline (dox). The scale bar corresponds to 200 μm. (**B**) Flow cytometry analysis of VE-Cad and CD41 expression after 2 days of culture in absence or presence of dox. (**C**) Bar graph representing the average frequency of the 2 indicated populations from 3 independent experiments. The p-values were calculated with Student’s t-test (2 tails, type 3). VE-Cad^+^ CD41^+^ population: **“No dox” versus “with Dox” p-value = 0.0002 (n = 3); VE-Cad^−^ CD41^+^ population: **“No dox” versus “with Dox” p-value = 0.0018 (n = 3). (**D**) Box plots comparing the expression of vascular specific genes between the 2 conditions. (**E**) Box plots comparing the expression of EMT specific genes between the 2 conditions. The box plots were generated from 3 independent experiments. For each plot, the top and bottom box edges correspond to the first and third quartiles. The black line inside the box represents the median. The top and bottom whisker lines mark the maximum and minimum values of the data set, respectively. The corresponding p-values were calculated with Student’s t-test (Supplementary Table S9).

We generated a dox-inducible Sox17-mCherry ES cell (iSox17) line in which addition of dox leads to the overexpression of the Sox17 protein (Supplementary Fig. S9A and S9B). We then sorted Pre-HPC generated from the iSox17 ES cell line and cultured the cells either with or without dox. Time-lapse microscopy showed a block in the formation of round cells (Fig. 7A, Supplementary Fig. S9C and Supplementary Videos S5 & S6) and in fact the majority of the cells still maintained the Pre-HPC phenotype (VE-Cad^+^ CD41^+^) 48 hours after the treatment with dox (Fig. 7B,C). Next, we extracted RNA from these cells and tested by q-RT-PCR the expression of endothelial and hematopoietic genes as well as the genes coding for the EMT markers. We showed that the expression of many endothelial genes was higher in presence of Sox17 (Fig. 7D and Supplementary Table S9). In contrast, the expression of *Spi1* and *Itgam* was clearly reduced. Interestingly *Runx1*, *Itga2b*, *Itgb3* and *Mpo* expression remained similar between the 2 conditions (Supplementary Fig. S9D and Supplementary Table S9). Finally the expression of most of the tested EMT genes did not change significantly at the exception of *Acta2* (Fig. 7E and Supplementary Table S9).

In conclusion, we showed that the overexpression of *Sox17* alone was enough to block the morphological changes occurring during EHT. However, we did not notice a decrease of CD41 expression and the scattered mesenchymal morphology observed after TGFβ treatment.

## Discussion

In this work, we used gain and loss of function approaches to study the impact of TGFβ signalling on the emergence of blood progenitors from endothelial cells with the model of *in vitro* ESC differentiation. Our working hypothesis was to consider that the EHT process would require similar signalling pathways as the ones involved in the EndMT, which occur during embryonic development and have been involved in several diseases^11^,^12^. We focused on the TGFβ pathway whose activity as an activator of EndMT and EMT is well documented^8^.

Our secretome analysis, the first one ever done on early blood development from mesodermal cells indicated that TGFβ treatment of BL-CFC differentiated cells for only 6 hours was enough to increase significantly the production of proteins involved in cell migration, vascular development and cell adhesion. The increase of MMP2 protein quantity in the extracellular space after TGFβ2 treatment is in line with reports linking TGFβ activity with MMP2 secretion and induction of EMT^8^,^29^,^39^. However we had no evidence of an enhancement of blood cell formation.

We next focused on the Pre-HPC population, which is the intermediary step between EC and HPC stages. Time-lapse imaging and flow cytometry experiments clearly demonstrated that TGFβ2 treatment inhibits haematopoiesis. Gene expression profiling further confirmed this as there was a dramatic reduction of genes related to the blood lineage. Finally, we detected the up-regulation of genes related to vasculature, extracellular matrix and cell adhesion, which was consistent with the proteomics data. We noticed also an increase of EMT markers^8^. That showed that not only TGFβ2 blocked haematopoiesis but also led to an EndMT process rather than EHT in our system.

ACVRL1 (ALK1) and TGFBR1 (ALK5) can both mediate TGFβ signalling in endothelial cells. Both receptors are expressed in EC and Pre-HPC (Fig. 1B). However our data suggest that the signalling through TGFBR1 is inhibiting blood cell formation and inducing EndMT. This is supported by a study^40^ showing that constitutive activation of ACVRL1 in EB culture increases the number of haematopoietic progenitors while a constitutive active form of TGFBR1 reduces haematopoietic differentiation. Whereas their work did not show exactly which cell type was affected by the activation of TGFBR1 activity, their results are consistent with our findings.

In line with our findings that inhibition of TFGβ signalling supports haematopoiesis, a study provided evidence that SB431542 treatment promotes the generation of a higher frequency of haematopoietic progenitors from human pluripotent stem cells^41^.

**Figure 8:**
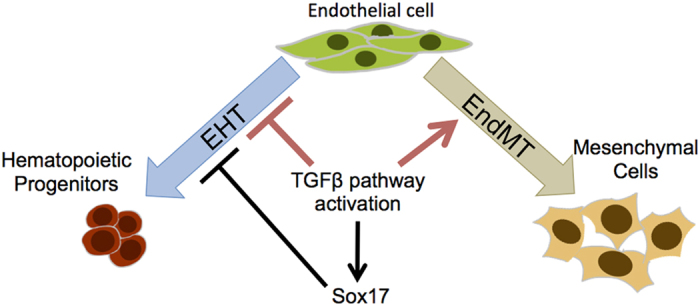
Model. The TGFβ signalling activation inhibits EHT through the up-regulation of Sox17 expression and the induction of EndMT.

To find out how TGFβ signalling activation blocks EHT, we chose to study the gene coding for the transcription factor Sox17. It has been proposed that *Sox17* is necessary for the formation of haemogenic endothelium *in vivo*^42^. A conditional knockout of *Sox17* in endothelial cells strongly abrogates haematopoietic stem cells formation in the mouse embryo^42^. In contrast, a recent publication by the group of Ann Zovein has shown that overexpression of *Sox17* in endothelial cells from AGM inhibits blood cell formation^43^. The discrepancy may come from the fact that *Sox17* is essential for arterial specification^38^. Haemogenic endothelial cells are of arterial type and therefore need *Sox17* to be produced. However once haemogenic endothelial cells are produced, *Sox17* has to be down-regulated to enable the EHT to take place and generate blood progenitor and stem cells. Our results support the findings of Lizama *et al.*^43^. The overexpression of *Sox17* blocked severely the morphological transition from endothelial to round blood cells. This was very similar to the effect of TGFβ activation. Nevertheless there were clear differences such as the lack of mesenchymal phenotype and the maintenance of CD41 expression. Apart from an increase of *Acta2* gene expression, most of the EMT marker genes were not transcribed at a higher level when Sox17 was up-regulated. We are proposing a model by which TGFβ induces expression of endothelial genes such as *Sox17*, which can block EHT. Other unidentified factors independent of *Sox17* may also play a role in inhibiting haematopoiesis and promoting the mesenchymal phenotype that we observed (Fig. 8).

Overall our study suggests that the TGFβ signalling through TGFBR1 has to be tightly regulated to not inhibit EHT and ensure an effective production of HPC. Our q-RT-PCR data on *in vivo* populations involved in EHT showed that EC and Pre-HPC are capable to respond to TGFβ signalling through TGFBR1. Our work could form the basis of new studies in the embryo to uncover how the TGFβ signalling affects the emergence of blood progenitors and stem cells.

Finally whereas our data suggest that EMT/EndMT and EHT are initiated by different processes, it would be worthwhile to test whether other signalling pathways involved in EMT such as EGF and PDGF pathways^44^,^45^ could play a role in EHT.

## Methods

### Embryonic stem cell growth and differentiation

The *Runx1*+^*/hCD4*^ 46, iGFP-Smad7 and iSox17-mCherry ES cell lines were used. Growth and differentiation of ES cells were performed according to previous protocols^47^. The ES cell culture media was made of Knockout™ DMEM (Life technologies, cat. #10829-018), 15% FBS (PAA, cat. #A15-102), 0.0024% 1 mg/ml LIF (EMBL-Heidelberg) and 0.24% 50 mM 2-mercapto-ethanol (Life technologies, cat. #31350-010).

### Mouse embryo generation

Timed mating of wild type C57BL/6-N mice were set up and the morning of vaginal plug detection was considered day 0.5. Day 11 embryos were staged by morphological landmarks. All experiments were performed in accordance with the guidelines and regulations defined by the European and Italian legislations (Directive 2010/63/EU and DLGS 26/2014, respectively). They apply to foetal forms of mammals as from the last third of their normal development (from day 14 of gestation in the mouse). They do not cover experiments done with day 11 mouse embryos. Therefore no experimental protocol or licence was required for the current study. Mice were bred and maintained at EMBL Monterotondo Mouse Facility in accordance with European and Italian legislations (EU Directive 634/2010 and DLGS 26/2014, respectively).

### Generation of doxycycline inducible ESC lines

The doxycycline-inducible GFP-Smad7 and Sox17-mCherry ESC lines were produced using the method of inducible cassette exchange described previously^48^. Briefly the GFP-Smad7 insert was excised from the pcDNA5-FRT-TO-GFP-SMAD7 plasmid while the Sox17-mCherry cDNA was synthesised by the GenScript Gene Synthesis Service (http://www.genscript.com/gene_synthesis.html). Both inserts were cloned into the p2Lox plasmid and subsequently transfected in A2Lox.cre ESC line previously treated with 0.5 μg/mL doxycycline for one day. Selection was performed in 300 μg/mL of G418 (Gibco) antibiotic for 10 days. Ten colonies were then picked and tested by flow cytometry for GFP or mCherry expression following a 24 h culture with and without doxycycline. The ESC clones presenting the highest expression level of fluorescent protein upon doxycycline induction were then chosen for further experiments.

### Flow cytometry and cell sorting

Staining was done as described previously^46^ and analyses were performed with a FACSCanto (Becton Dickinson). Sorts were performed with a FACSAria (Becton Dickinson) or by using magnetic sorting (Miltenyi Biotec, cat. #130-090-485) and anti-APC microbeads (Miltenyi Biotech, cat. #130-090-855). Monoclonal rat anti-mouse antibodies used were Anti-Mouse CD309 (FLK1) APC (Avas12a1, eBioscience, cat. #17-5821-81), Anti-Mouse CD41 PE (MWReg30, eBioscience, cat. #12-0411-81), Anti-Mouse CD144 (VE-Cadherin) eFluor® 660 (eBioBV13, eBioscience, cat. #50-1441-80). FACS data were analysed using the FlowJo software (Tree Star, Inc.).

### BL-CFC and haemogenic endothelium cultures

Flk-1^+^ cells from day 3–3.25 EBs were cultured on gelatinized plate at a density of 0.1 × 10^6^ cells per cm^2^ in the BL-CFC differentiation media containing IMDM supplemented with 10% FBS (PAA Clone, cat. #A15–102), 1% L-glutamine (GIBCO, cat. #25030-024), 0.6% 30 mg/ml transferrin (Roche Diagnostics Limited, cat. #10652202001), 0.3% 0.15 M Monothioglycerol (MTG) (Sigma, cat. #M6145-25 ml), 0.5% 5 mg/ml ascorbic acid (Sigma, cat. #A-4544), 15% D4T conditioned medium, 0.1% 5 μg/ml VEGF (R&D systems, cat. #293-VE), and 0.1% 10 μg/ml IL-6 (R&D systems, cat. #406-ML).

For haemogenic endothelium culture, FACS-sorted VE-Cad^+^CD41^+^ cells (Pre-HPC) were cultured on gelatinized plate at a density of 0.2 × 10^6 ^cells per cm^2^ (cells isolated from day 1.5 BL-CFC culture). The medium was composed of IMDM supplemented with 10% FBS (same as BL-CFC mix), 1% L-glutamine, 0.6% 30 mg/ml transferrin, 0.3% 0.15 M MTG, 0.5% 5 mg/ml ascorbic acid, 0.024% 100 μg/ml LIF (EMBL-Heidelberg), 0.5% 10 μg/ml SCF (R&D systems, cat. #455-MC), and 0.1% 10 μg/ml Oncostatin M (R&D systems, cat. #495-MO). Cultures were maintained in a humidified chamber in a normal O_2_ 5% CO_2_-air mixture at 37 °C.

### Quantitative RT-PCR

Two different methods were used for quantitative RT-PCR depending on the number of cells used for analysis.

For number of cells ranging from 0.1 × 10^6^ to 1 × 10^6^, RNA extraction was done using either the QIAGEN RNeasy Micro Kit (Qiagen, cat. #74004) or Qiagen RNAeasy Plus Mini kit (Qiagen, cat. #74134). The cDNA was synthesized using the RevertAid H minus First Strand cDNA synthesis kit (ThermoFisher Scientific, cat. #K1631). Random hexamer primers (0.2 μg) were added to total RNA (500 ng), and the mix was incubated at 65 °C for 5 min. Reaction buffer (1x), RiboLock RNase inhibitor (20U), dNTP mix and RevertAid Reverse transcriptase (200U) were added to the mixture and mixed gently. The reaction mix was then incubated at 25 °C for 5 min followed by an incubation at 42 °C for 60 min. Reaction was terminated by a 70 °C incubation for 5 min. Quantitative PCR reactions (see Supplementary Table S10 for primer sequences) were performed with the LightCycler 480 SYBR Green I Master kit (Roche, cat. #04707516001) and the Roche LightCycler 480 RT-PCR instrument. The PCR results were normalised according to the reference gene *Ppia*^49^.

For 25 cells we used a nested PCR approach with the Fluidigm Biomark HD system. Nested PCR was done for each gene with 2 outer primers and 2 inner primers (see Supplementary Table S11 for primer sequences) according to the Fluidigm Advanced Development Protocol, section 41. Reagents of the CellsDirect One-Step qRT-PCR kit (Invitrogen cat. #11753) were used for this protocol. Twenty-five cells of EC, Pre-HPC and HPC populations from *in vitro* differentiated ESC or cells isolated from E11 AGM region were FACS-sorted in one well of 96-well plate (Bio-Rad Hard-Shell PCR plates cat. #HSP9611) filled with 5 μl of 2x reaction mix (Invitrogen cat. #11753). The cells were snap-frozen immediately on dry ice after sort and stored at −80 °C. RT/Specific Target amplification (STA) was performed with a mix composed of 2.8 μl of resuspension buffer (Invitrogen cat. #11753), 0.2 μl of SuperScriptIII & Platinum Taq polymerase (Invitrogen cat. #11753) and 1 μl of 500 nM outer primer mix (forward and reverse primers). Four μl of the RT-STA mix were added to the frozen cells and the RT-STA proceeded according to the following steps. The RT were done with a 50 °C incubation for 15 minutes, followed by inactivation at 95 °C for 2 minutes. Then the STA reaction were performed at 95 °C for 15 seconds and 60 °C for 4 minutes for 20 cycles. The cDNA within each well was diluted 1/5 and the sample mix was prepared with loading reagent (Fluidigm) and SsoFast™ EvaGreen® Supermix (Bio-Rad). In parallel individual assay mixes were prepared as 5 μM inner primer mix, with DNA suspension buffer and assay loading reagent. After the priming of 96.96 dynamic array IFC (Fluidigm, cat. #BMK-M-96.96), the sample mix and assay mixes were put together in the corresponding inlets and loaded into the chip. Finally the chip was run in the Biomark by using the Biomark Data Collection Software and the GE96 × 96PCR+Meltv2.pcl program. Raw data were opened and analysed with the Fluidigm Real Time PCR Analysis software. For each primer, Ct values and melting curves could be visualized. The raw data were analysed with the following options: quality threshold set at 0.65, Ct threshold was Auto (Global), and baseline correction method was linear (derivative). The whole experiment was performed 3 to 4 times. The PCR results were normalised according to the reference gene *Ppia*.

Quantitative RT-PCR expression results were displayed as box plots with the function boxplot in R (http://www.r-project.org).

### Activation and inhibition of the TGFβ pathway

To activate the TGFβ pathway, the recombinant mouse TGFβ2 (R&D systems, cat. #7346-B2, re-suspended in 4 mM HCl+0.1% BSA 1X PBS) was added into the culture medium at 5 ng/ml final concentration. The inhibition of the TGFβ pathway was performed using SB431542 (Tocris, cat. #1614, re-suspended in DMSO) at 10μM final concentration. Vehicle controls for TGFβ2 and SB431542 were the same volumes of 4 mM HCl+0.1% BSA 1X PBS and DMSO, respectively.

### Proteomics analysis of secreted proteins

After 24 hours of BL-CFC culture the medium was removed and cells were washed with pre-warmed 1X PBS twice. Then they were incubated with an adapted BL-CFC culture medium made of DMEM depleted of methionine, arginine and lysine (Gibco) and 10% dialyzed FBS (Gibco) which was supplemented with 0.1 mM L-AHA (AnaSpec, Inc) and either 84 μg/ml [^13^C_6_] L-arginine and 146 μg/ml [d_4_] L-lysine or 84 μg/ml [^13^C_6_^15^N_4_] L-arginine and 146 μg/ml [^13^C_6_^15^N_2_] L-lysine (Cambridge Isotope Laboratories, Inc). Cells were treated either with TGFβ2 or vehicle control for 6 hours. The supernatant was taken, spun to remove floating cells and frozen on dry ice. The enrichment of newly made proteins, LC-MS/MS and data analysis were performed as described^22^. A total of 3 independent experiments were performed. Visual representation of identified proteins and gene ontology analysis were done with STRING 10 (http://string-db.org)^23^.

### Time-lapse photography

The phase contrast time-lapse images were taken with the IncuCyte HD (Essen Biosciences) inside an incubator, every 15 minutes, 9 areas per well. The time-lapse videos have 10 frames per second. They were made with the Fiji software (http://fiji.sc/Fiji). The images from each area were analysed by the Cell Profiler software (http://www.cellprofiler.org) to quantify the number of round cells in each frame with a customized pipeline written by Christian Tischer from the EMBL Heidelberg Advanced Light Microscopy Facility. For each time point, the average and standard deviation of counts from all 9 spots were calculated to make graph with Microsoft Office Excel software.

### mRNA microarray

Total RNA extracted with Qiagen RNAeasy Plus Mini kit (Qiagen, cat. #74134) was tested for its quality with BioAnalyzer (Agilent) and its concentration measured using Qubit (Life Technologies). The samples were hybridised on Affymetrix GeneChip Mouse Gene 2.0 ST Array. Data were analysed using R with the packages limma, oligo, pvclust, genefilter and pd.mogene.2.0.st. Gene Ontology studies were performed with the Database for Annotation, Visualization and Integrated Discovery (DAVID) tool (http://david.abcc.ncifcrf.gov).

## Additional Information

**How to cite this article**: Vargel, Ö *et al.* Activation of the TGFβ pathway impairs endothelial to haematopoietic transition. *Sci. Rep.* **6**, 21518; doi: 10.1038/srep21518 (2016).

## Supplementary Material

Supplementary Information

Supplementary Video S1

Supplementary Video S2

Supplementary Video S3

Supplementary Video S4

Supplementary Video S5

Supplementary Video S6

Supplementary Table S2

Supplementary Table S4

Supplementary Table S5

Supplementary Table S6
