# Supplementary material for: Activation of the TGFβ pathway impairs endothelial to haematopoietic transition
Source: Sci Rep. 2016 Feb 19;6:21518. doi: 10.1038/srep21518 (PMC4759586; doi:10.1038/srep21518)
Supplement: Supplementary Information [file srep21518-s1.pdf]

Supplementary Information

Activation of the TGFβ pathway impairs endothelial to haematopoietic transition

Özge Vargel[1], Yang Zhang[1,4], Kinga Kosim[1], Kerstin Ganter[1], Sophia Foehr[2], Yannicka Mardenborough[1], Maya Shvartsman[1], Anton J. Enright[3], Jeroen Krijgsveld[2] & Christophe Lancrin[1,5]

[1] European Molecular Biology Laboratory, Mouse Biology Unit, Via Ercole Ramarini 32, 00015 Monterotondo, Italy
[2] European Molecular Biology Laboratory, Genome Biology Unit, Meyerhofstraße 1, 69117 Heidelberg, Germany
[3] European Molecular Biology Laboratory, European Bioinformatics Institute, Wellcome Genome Campus, Hinxton, Cambridge, CB10 1SD, United Kingdom.
[4] Present address: Uppsala Universitet, Rudbecklaboratoriet, 751 85 Uppsala, Sweden
[5] Correspondence should be addressed to christophe.lancrin@embl.it
Phone: +39 06 90091 218

Supplementary Figure S1: Analysis of the gene expression during in vitro EHT

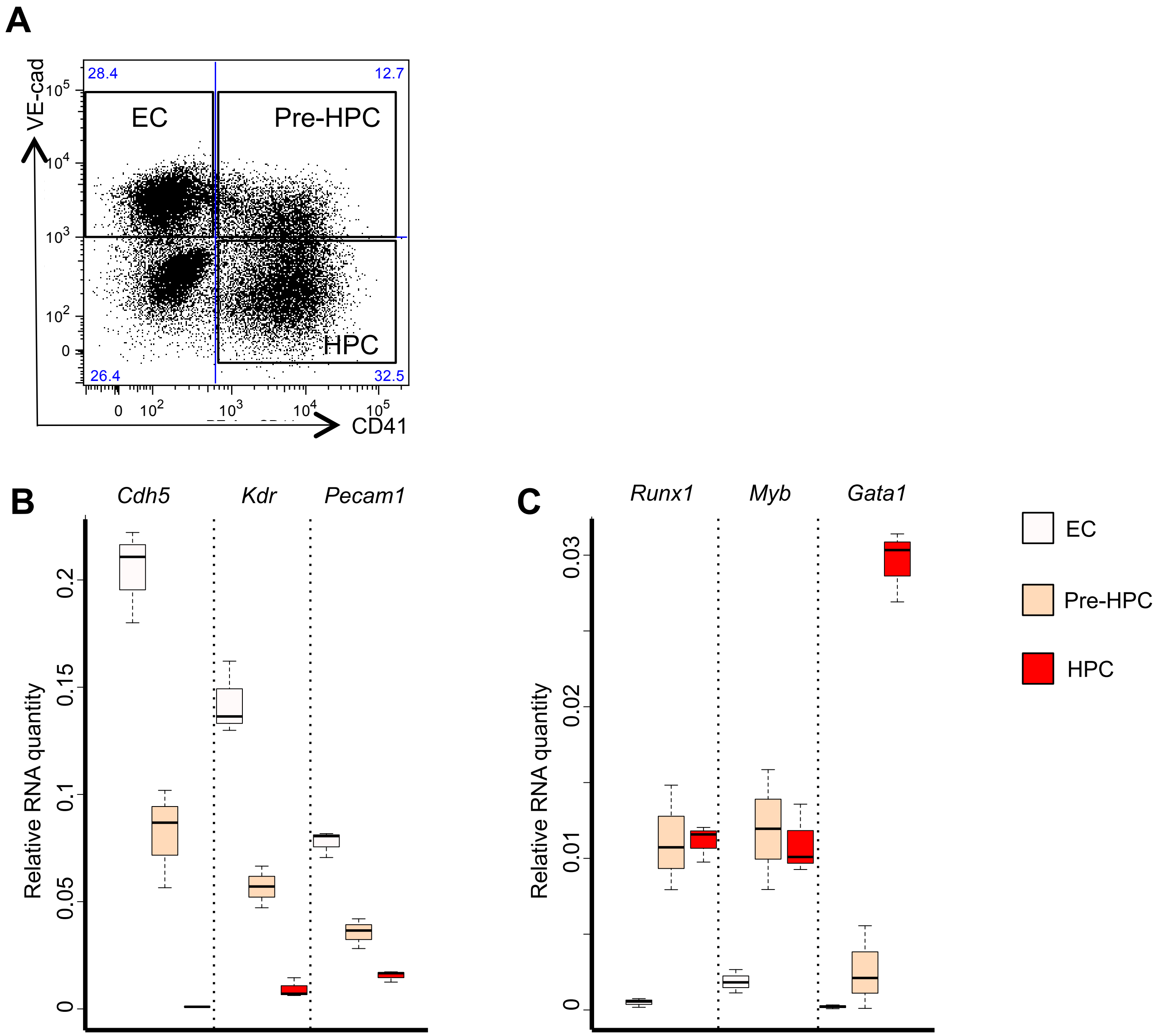

Supplementary Figure S2: Analysis of the secretome LC-MS/MS data

A

	Protein identified	Protein identified in normalized replicates	Proteins with secreted evidence	Protein quantified
**Replicate 1**	133	119	80	119
**Replicate 2**	169	164	92	164
**Replicate 3**	268	179	100	178

B

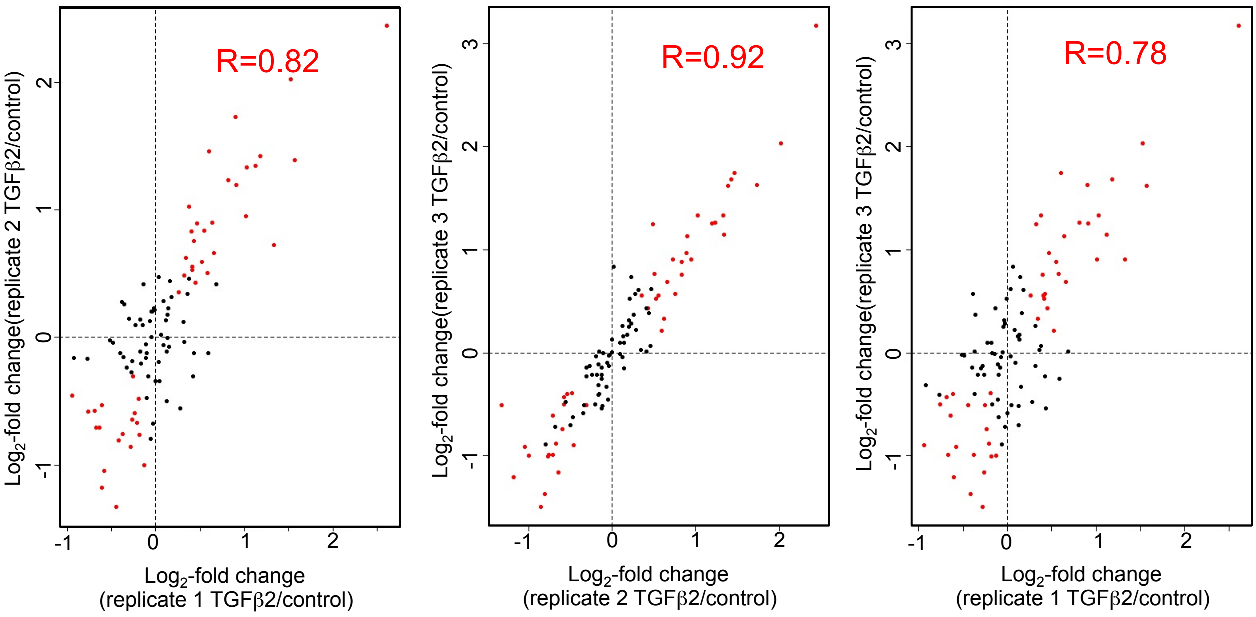

Supplementary Figure S3: Gene ontology (GO) analysis of the genes coding for the 33 secreted proteins detected in all 3 biological replicates

GO:0030334: Regulation of Cell Migration

12 genes, p-value bonferroni= 2.06E-07

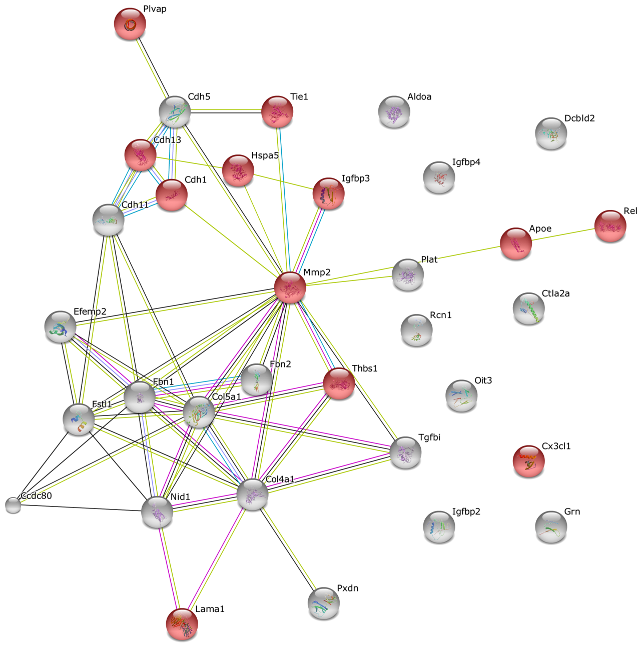

GO:0030335: Positive regulation of cell migration

7 genes, p-value bonferroni= 7.14E-03

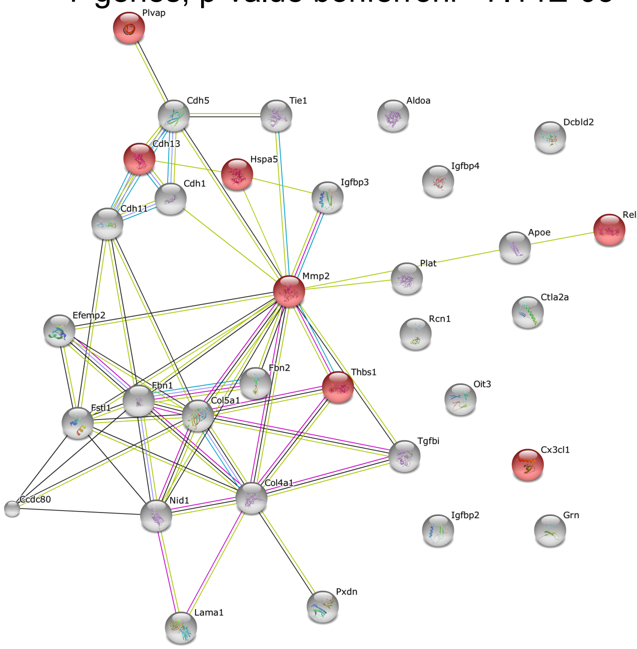

GO:0030198: Extracellular matrix organisation

6 genes, p-value bonferroni= 2.78E-03

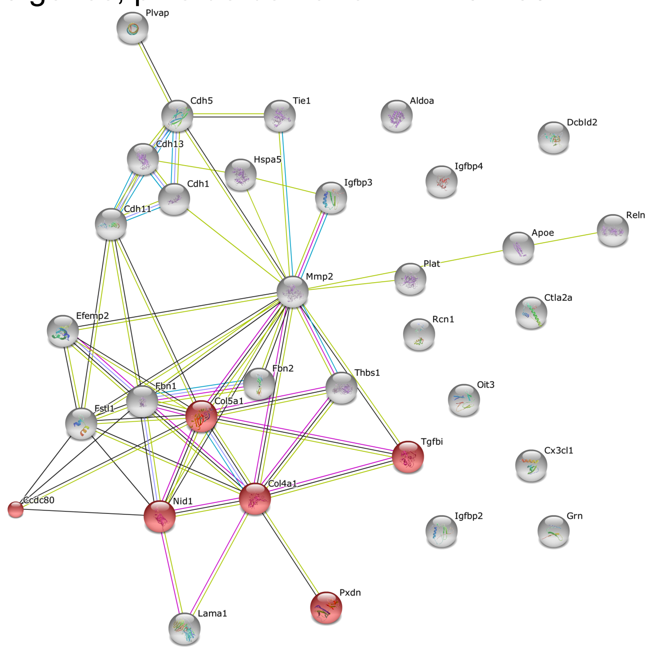

GO:001944: Vasculature development

7 genes, p-value bonferroni= 3.36E-02

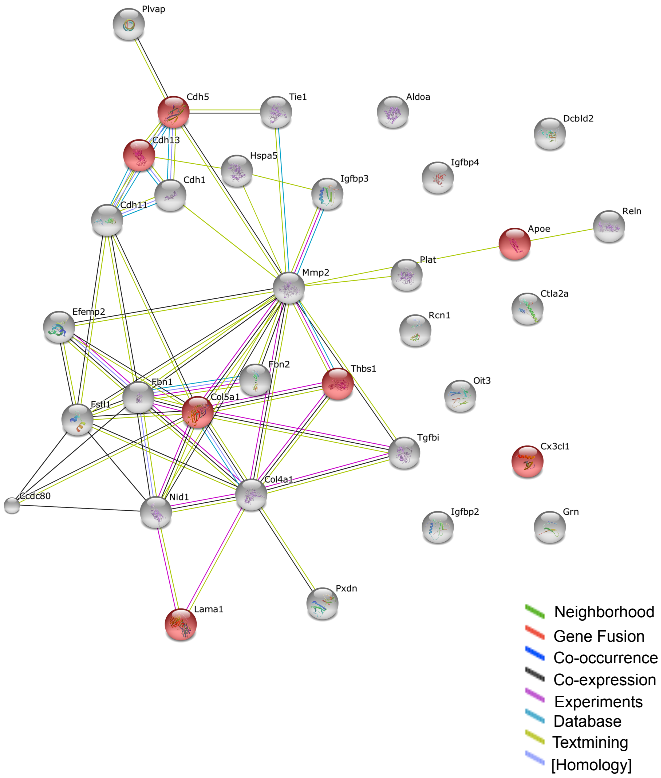

Supplementary Figure S4: **Experimental layout for the experiments described in Figures 3 and 4**

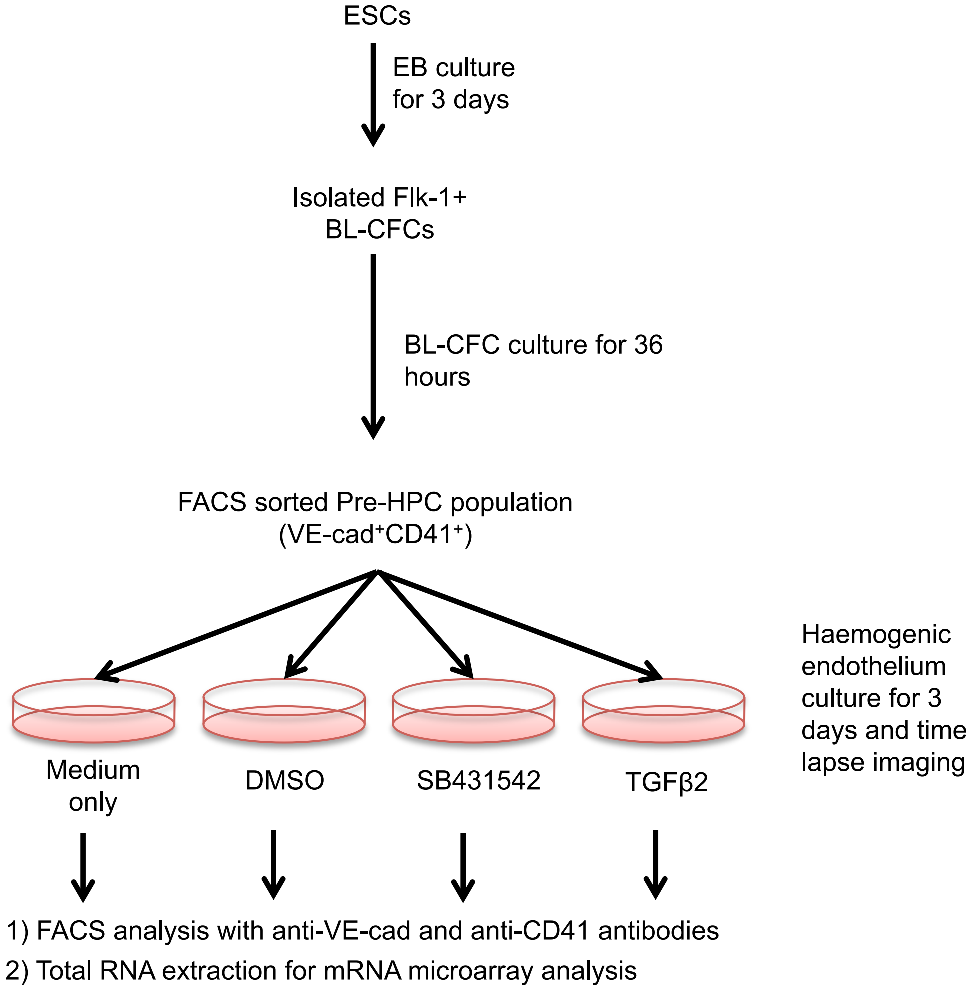

Supplementary Figure S5: Inhibition of the TGFβ pathway with a dox inducible GFP-Smad7 ESC line

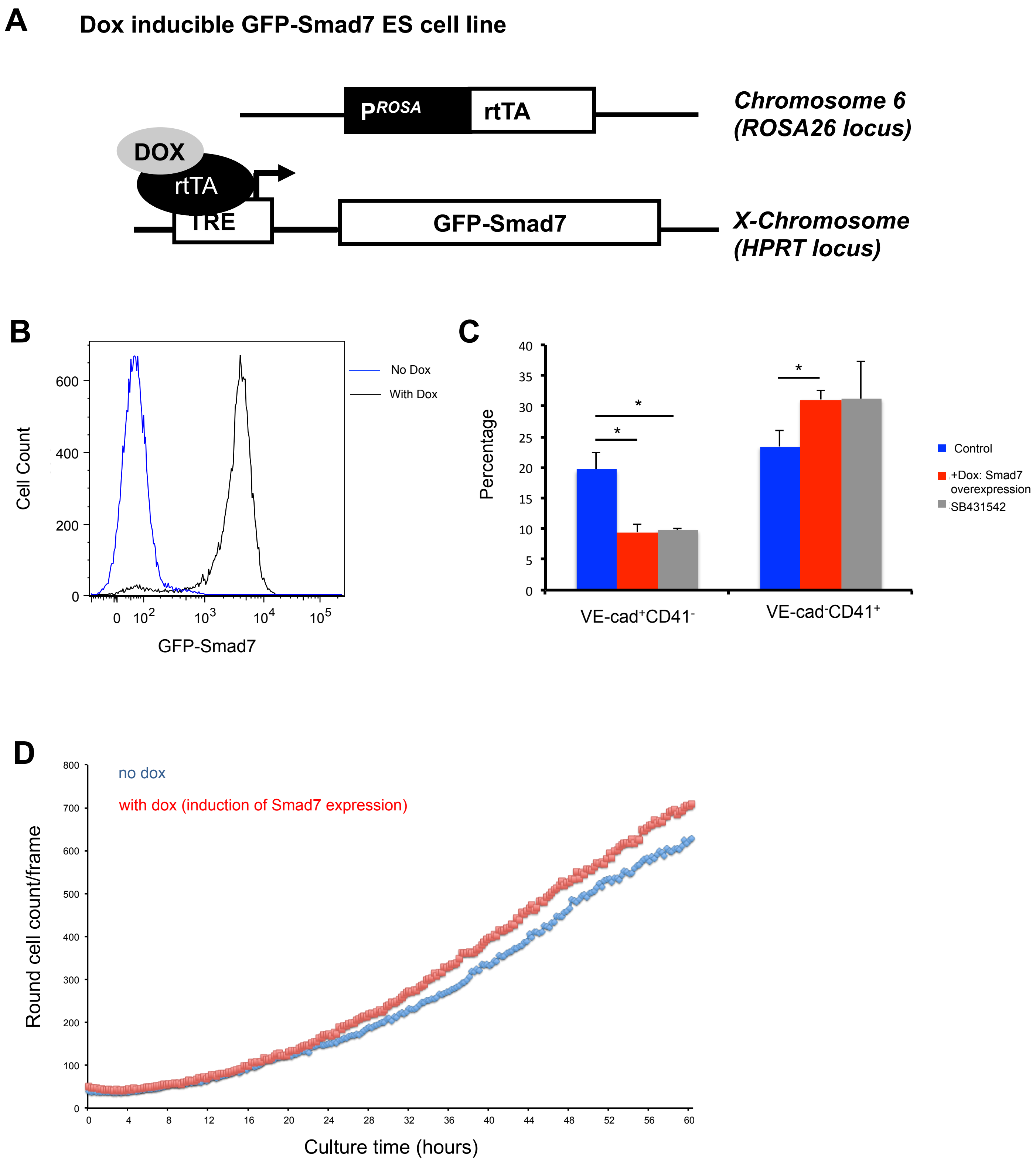

Supplementary Figure S6: **Heatmap of gene expression from microarray analysis of Figure 4**

A

	Control (DMSO)	Inhibitor (SB431542)	Activator (TGFβ2)
replicate 1	Con_mOV1	Inh_mOV2	Act_mOV3
replicate 2	Con_mOV4	Inh_mOV5	Act_mOV6
replicate 3	Con_mOV7	Inh_mOV8	Act_mOV9

B

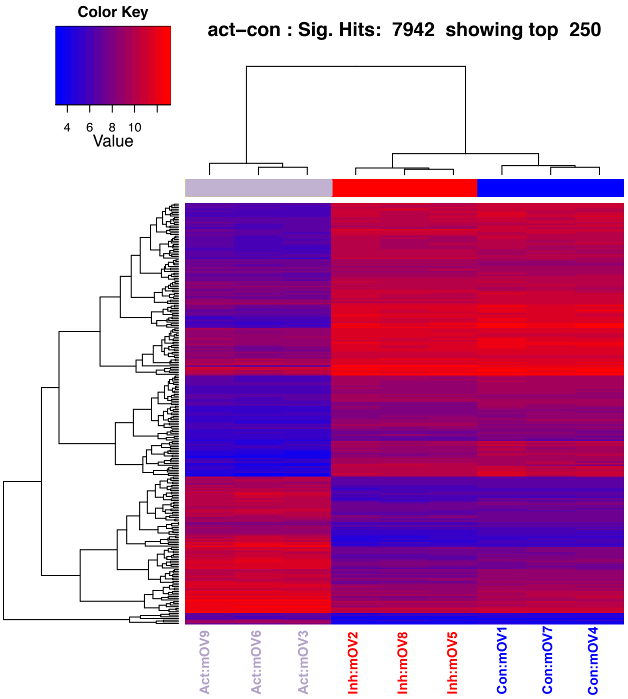

Supplementary Figure S7: Analysis of the gene expression during in vivo EHT

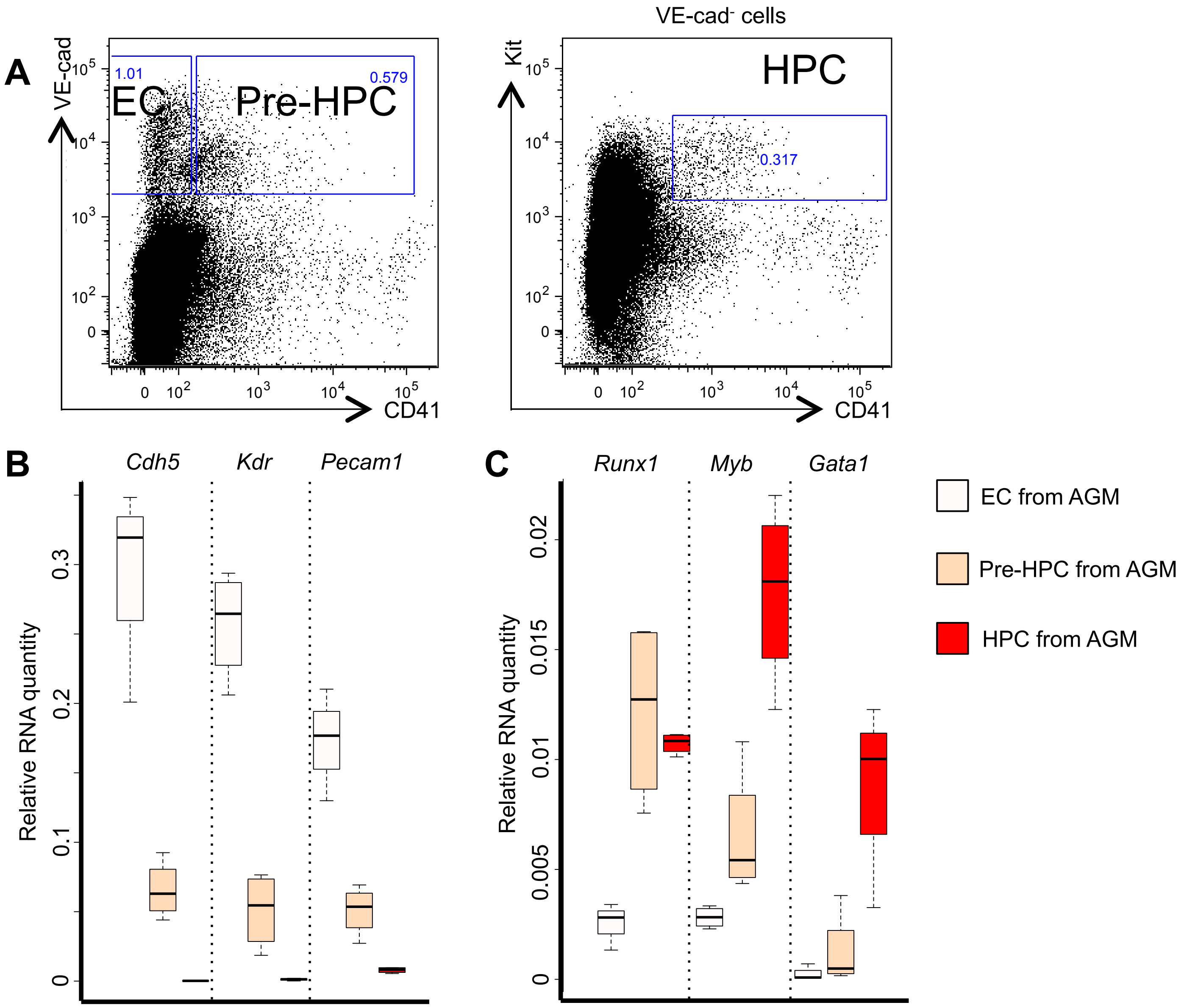

Supplementary Figure S8: **q-RT-PCR validation of genes found differentially expressed after activation of the TGFβ pathway**

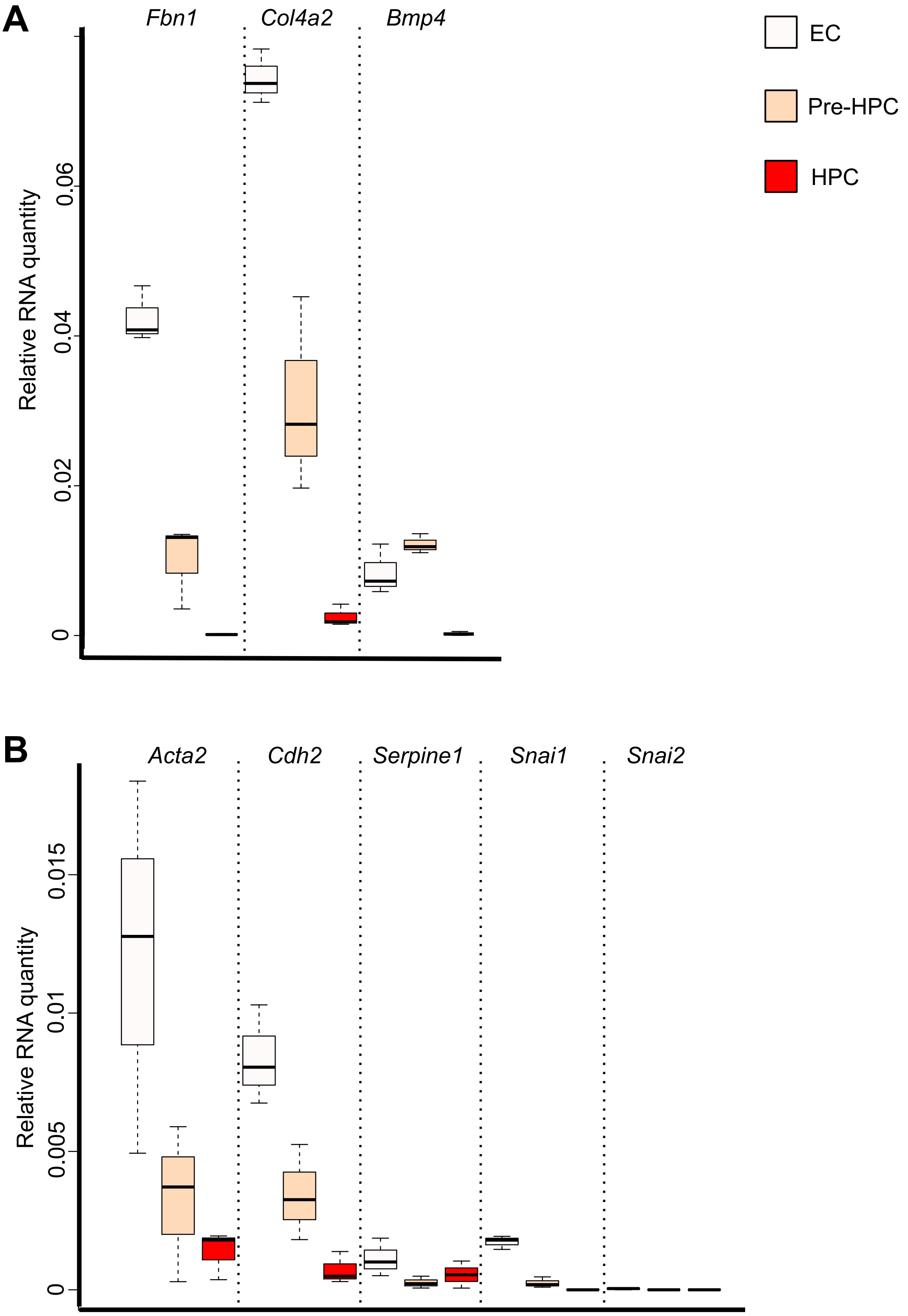

Supplementary Figure S9: **Characterisation of the dox inducible Sox17-mCherry ES cell line**

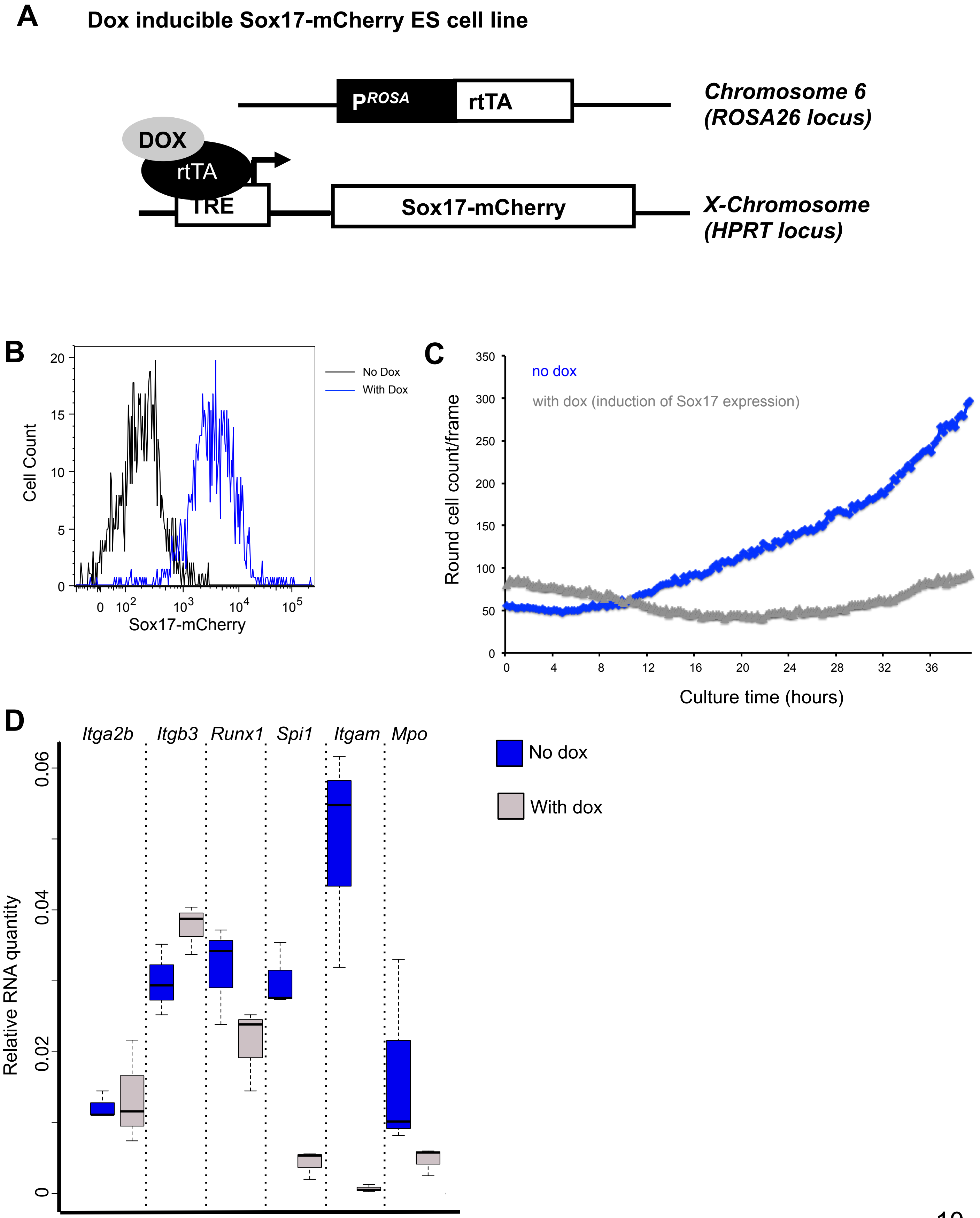

Supplementary Table S1: P-values from T-test for the q-RT-PCR results shown in Figure 1, Supplementary Figure S1 and Supplementary Figure S8

Gene names	P-value EC versus HPC	P-value EC versus Pre-HPC	P-value Pre-HPC versus HPC
Acvrl1	0,015122719	0,014924923	0,065488053
Eng	0,004527772	0,016756591	0,015577393
Tgfbr1	0,453636233	0,26105109	0,032650805
Tgfbr2	0,01534493	0,021924717	0,006535239
Tgfb1	0,09731877	0,002163498	0,345030705
Tgfb2	0,022909347	0,021155441	0,211072248
Tgfb3	0,422399561	0,89892691	0,422656718
Smad1	0,453636233	0,26105109	0,032650805
Smad2	0,118587101	0,01094977	0,933824552
Smad3	0,009277296	0,072926113	0,03876289
Smad4	0,00730037	0,019237812	0,003893865
Smad5	0,453636233	0,26105109	0,032650805
Smad6	0,009009313	0,133684139	0,064690344
Smad7	0,428871174	0,065857651	0,154733242
Smad8	0,188220027	0,190874843	0,478088182
Cdh5	0,003799599	0,002638116	0,026222623
Kdr	0,003383252	0,003923056	0,005496671
Pecam1	0.000927057	0.001526393	0.026487105
Runx1	0.002841714	0.032671344	0.986481783
Myb	0.013214429	0.043506915	0.743727752
Gata1	0.002068419	0.273835643	0.000242192
Fbn1	0.002579086	0.002079356	0.093266515
Col4a2	0.000170282	0.022195802	0.061063367
Bmp4	0.050209525	0.183093346	0.002919927
Acta2	0.109469223	0.141667469	0.358899073
Cdh2	0.01169988	0.026771959	0.10032369
Serpine1	0.305457146	0.150559583	0.425213466
Snai1	0.0064278	0.001450526	0.16198896
Snai2	0.230559599	0.205096195	0.428115328

Supplementary Table S3: **Log$_2$ fold change TGFβ2/control for the 33 proteins detected in the all 3 biological replicates**

Protein names	Log$_2$-fold change TGFβ2/control replicate 1	Log$_2$-fold change TGFβ2/control replicate 2	Log$_2$-fold change TGFβ2/control replicate 3	p-value
IGFBP3	2.61	2.51	3.26	2.29E-47
DCBLD2	1.52	2.09	2.12	7.97658E-27
COL5A1	0.90	1.80	1.71	1.51459E-17
CDH1	0.60	1.53	1.83	1.101E-14
CDH5	1.18	1.49	1.77	1.15045E-17
PLVAP	1.57	1.46	1.71	1.26422E-19
CX3CL1	1.12	1.41	1.23	1.68257E-13
APOE	1.02	1.40	1.42	5.521E-14
FBN1	0.81	1.30	1.35	9.39535E-12
NID1	0.90	1.26	1.34	5.57186E-12
CCDC80	0.37	1.09	1.42	1.24895E-08
MMP2	0.64	0.97	1.22	2.3726E-08
LAMA1	0.54	0.91	0.96	1.75234E-06
FBN2	0.40	0.90	0.85	2.22078E-05
CDH11	1.33	0.79	0.99	8.7393E-10
TIE1	0.66	0.73	0.78	1.88091E-05
PXDN	0.34	0.69	0.42	0.004336367
PLAT	0.41	0.62	0.64	0.001012252
COL4A1; COL4A5	0.41	0.60	0.61	0.001450587
FSTL1	0.58	0.57	0.85	7.52462E-05
IGFBP2	0.32	0.55	1.33	1.24017E-05
THBS1	0.03	0.54	0.71	0.012726367
EFEMP2	0.16	0.51	0.47	0.02475059
GRN	0.45	0.50	0.52	0.003797814
CTLA2A	0.26	0.42	0.64	0.009632005
CDH13	0.18	0.39	0.69	0.013435941
TGFBI	0.14	0.29	0.82	0.013652648
IGFBP4	0.06	0.09	0.92	0.03650901
RELN	-0.94	-0.38	-0.80	2.62996E-05
RCN1	-0.61	-0.46	-0.30	0.007098022
OIT3	-0.67	-0.63	-0.89	1.41832E-05
HSPA5	-0.64	-0.63	-0.52	0.00041846
ALDOA; ALDOART1; ALDOART2	-0.58	-0.97	-0.82	2.46155E-06

Supplementary Table S7: P-values for the q-RT-PCR results shown in Figure 5

Gene names	P-value Control versus TGFβ2	P-value SB431542 versus TGFβ2	P-value Control versus SB431542
Cdh5	0.000601231	0.000498666	0.04218058
Pecam1	0.002169357	0.000587166	0.236798344
Bmp4	0.003178243	0.001150749	0.040429672
Col4a2	0.010809986	0.008237105	0.101433008
Fbn1	0.013960292	0.013496887	0.054798199
Sox17	0.001567875	0.001173283	0.12962107
Itga2b	0.005457093	0.000570812	0.054396316
Itgb3	0.356631661	0.029066075	0.037717558
Runx1	0.009096043	0.006413325	0.453531279
Spi1	0.000117523	0.000388597	0.003306173
Itgam	0.013438532	0.035413337	0.220825924
Mpo	2.2498E-06	0.007585923	0.015813011
Acta2	0.036711979	0.034167917	0.020543456
Cdh2	0.013424011	0.013248324	0.132819548
Serpine1	0.061966143	0.06064261	0.00069959
Snai1	0.0096596	0.007423555	0.190497012
Snai2	0.04646809	0.00661859	0.186100512

Supplementary Table S8: P-values from T-test for the q-RT-PCR results shown in Figure 6 and Supplementary Figure S7

Gene names	P-value EC versus HPC	P-value EC versus Pre-HPC	P-value Pre-HPC versus HPC
Acvrl1	0.007301625	0.014777168	0.006406575
Eng	0.000544728	0.000224291	0.010328306
Tgfbr1	0.013946823	0.057212701	0.018535263
Tgfbr2	1.85867E-05	0.21049191	0.028151668
Tgfb1	0.555196088	0.011314105	0.421770725
Tgfb2	0.045932294	0.011935524	0.305931811
Tgfb3	0.014324767	0.899447102	0.016505044
Smad1	0.00167632	0.003091762	0.021555758
Smad2	0.008581348	0.021762272	0.963477548
Smad3	0.44404424	0.403666966	0.795334814
Smad4	3.69161E-05	0.00011774	0.051509425
Smad5	0.000914994	0.001377122	0.010731207
Smad6	0.14571663	0.780224458	0.145586198
Smad7	0.022106838	0.046677964	0.108912999
Smad8	0.110604282	0.518634473	0.045656939
Cdh5	0.002836704	0.00370604	0.007837204
Kdr	0.000947962	0.000233577	0.035474428
Pecam1	0.002061151	0.001721997	0.016056901
Runx1	3.19975E-05	0.017309801	0.534575712
Myb	0.005195313	0.085956116	0.005857585
Gata1	0.020835798	0.327897196	0.021798467
Fbn1	0.030600985	0.111057577	0.172408516
Col4a2	0.002912624	0.007861248	0.044104983
Bmp4	0.019646627	0.007861248	0.008552767
Acta2	0.982483569	0.146119415	0.39097696
Cdh2	0.108558196	0.527564852	0.810091517
Serpine1	0.143340269	0.52482827	0.375204974
Snai1	0.006207036	0.004882306	0.392885263
Snai2	0.377896203	0.002059913	0.250808108

Supplementary Table S9: P-values for the q-RT-PCR results shown in Figure 7 and Supplementary Figure S9

Gene names	P-value -dox versus +dox
Cdh5	0.03944808
Pecam1	0.065277133
Bmp4	0.387621631
Col4a2	0.021721996
Fbn1	0.03914846
Sox17	0.001050284
Itga2b	0.78785187
Itgb3	0.101500156
Runx1	0.117364612
Spi1	0.004155336
Itgam	0.032242612
Mpo	0.259830846
Acta2	0.026490028
Cdh2	0.067042239
Serpine1	0.238021224
Snai1	0.762343399
Snai2	0.184958342

Supplementary Table S10: List of primers used for q-RT-PCR done in Figure 5, Figure 7 and Supplementary Figure S9

Gene name	Forward	Reverse
Cdh5	TCATCAAACCCACGAAGTCC	GGTCTGTGGCCTCAATGTAGA
Pecam1	GCTGGTGCTCTATGCAAGC	ATGGATGCTGTTGATGGTGA
Bmp4	CAGCCGAGCCAACACTGTGA	TGGGATGCTGCTGAGGTTGA
Col4a2	GCGCACAACCAGGACCTA	TACAGGAAAGGCATGGTGCT
Fbn1	CAGATCCATCCAACACTGC	TACCCTTTCTGGCACAGAC
Sox17	CACAACGCAGAGCTAAGCAA	CGCTTCTCTGCCAAGGTC
Itga2b	TTCCAACCAGCGCTTCACCT	TGCTCGGATCCCCATCAAAC
Itgb3	TCCTCCAGCTCATTGTTGATGC	AGGCAGGTGGCATTGAAGGA
Runx1	CTCCGTGCTACCCACTCACT	ATGACGGTGACCAGAGTGC
Spi1	GGGATCTGACCAACCTGGA	AACCAAGTCATCCGATGGAG
Itgam	TACAGCACAAGCCGGTGTC	GGACAGGCCCAAGGACATA
Mpo	TACATGTGGCCCTAGACCT	GCAGGTGTCAACACATCTG
Acta2	AGGCACCACTGAACCCTAAG	CACAGCCTGAATAGCCACAT
Cdh2	ATCAACAATGAGACTGGGGACATCA	CTTCCATGTCTGTGGCTTGAA
Serpine1	AAAACCCGGCGGCAGATCCA	CTTGTTCCACGGCCCCATGA
Snai1	GAAGATGCACATCCGAAGC	GAATGGCTTCTCACCAGTG
Snai2	GACACATTAGAACTCACACTGG	AAAGCCCTATTGCAGTGAG

Supplementary Table S11: **List of primers used for q-RT-PCR done in Figure 1, Figure 6, Supplementary Figure S7 and Supplementary Figure S8**

Gene name	Outer forward	Outer reverse	Inner forward	Inner reverse
Acvrl1	CGAATTGCCCATCGTGACCTCAA	CGTGGTTGTTGCCGATATCCAGGTA	CGAAGTCGCAATGTGCTGGTCAA	CGAAGTCGCAATGTGCTGGTCAA
Eng	GCCTGACTTTCTGGGACTCCAGC	TGCTGACCACATGGGCTGTCAC	GCCAGGCTGAAGACACTGACG	TCATGCCGCAGCTGGAGTAG
Tgfbr1	GCAGACTTGGGACTTGCTGTGA	CATCTAGAACTTCAGGGGCCATGT	CATGATTCTGCCACAGATACAA	CCTTTTAGTGCCTACTCTGTGGTTTGG
Tgfbr2	TGGCCGCTGCATATCGTCCT	GCATCTTTCTGGGCTTCCATTTCCA	TGGACGCGCATCGCCAGCA	ATCCGACTTGGGAACGTG
Tgfb1	ACCCCCACTGATACGCCTGA	GCAGTGAGCGCTGAATCGAA	TGGCTGTCTTTTGACGTCACTG	GCCCTGTATTCCGTCTCCTTGG
Tgfb2	GGCATGCCCATATCTATGGAGTTC	CAGATCCTGGGACACACAGCA	GACACTCAACACACCAAAGTCCTCA	GGGAAGCGGAAGCTTCGGGATTTA
Tgfb3	CATGTCACACCTTTCAGCCCAAT	CTCCACGGCCATGGTCATCT	GAGACATACTGGAAAATGTTCATGAGGTG	CATTGTCCACTCCTTTGAATTTGA
Smad1	TCTCAGCCCATGGACACGAA	CACCAGTGTTTTGGTTCCTCGT	ATGATGGCGCCTCCACTGC	GCAACTGCCTGAACATCTCCTC
Smad2	TGCTCTCCAACGTTAACCGAAA	TCAGCAAACACTTCCCCACCT	GCCACTGTAGAAATGACAAGAAGACA	TGTAATACAAGCGCACTCCCCTTC
Smad3	CCAATGTCAACCGGAATGCAG	TGAGGCACTCCGCAAAGACC	CGTGGAACTTACAAGGCGACA	CCCCTCCGATGTAGTAGAGC
Smad4	TTGCCTCACCACCAAAACG	TGGAATGCAAGCTCATTGTGA	CCATCTTCAGCACCACCCGCCTA	TGGCCAGTAATGTCCAGGATG
Smad5	AACCATGGATTCGAGGCTGTG	TGACGTCCTGTCGGTGGTACTC	TGAGCTCACCAAGATGTGTACC	GCTCCCCAGCCCTTGACAAA
Smad6	TTCTCGGCTGTCTCCTCCTGAC	TTCACCCGGAGCAGTGATGA	GTACAAGCCACTGGATCTGTCCGATT	GGAGTTGGTGGCCTCGGTTT
Smad7	GGAAGATCAACCCCGAGCTG	TGAGAAAATCCATTGGGTATCTGGA	TGTGCTGCAACCCCCATCAC	AAGGAGGAGGGGGAGACTCTA
Smad8	GCACGATTCGGATGAGCTTTG	TGCAGCGGTCCATGAAGATG	GAAGGGCTGGGGAGCAGAGT	TCTCGATCCAGCAGGGGGTGCT
Cdh5	CGACACCATCGCCAAAAGAGAGAC	CGTCTTAGCATTCTGGCGGTTCAC	CGAGGATTTGGAATCAAATGCACATCG	CGAGGATTTGGAATCAAATGCACATCG
Kdr	TGTGGGGCTTGATTTCACCTG	TCGCCACAGTCCCAGGAAAG	CACTCTCCACCTTCAAAGTCTCATCA	TTTCACATCCCGGTTTACAATCTTC
Pecam1	TGCGGTGGTTGTCATTGGAG	CTGGACATCTCCACGGGTTT	GTCATCGCCACCTTAATAGTTGCAG	TGTTTGGCCTTGGCTTTCCTC
Runx1	CGAACTACTCGGCAGAACTGAGAA	CGTACGGTGATGGTCAGAGTGAA	CGAATGCTACCGCGGCCATG	CGAATGCTACCGCGGCCATG
Myb	CGAGTGGCAGAAAGTGCTGAACC	CGTTGCTTGGCAATAACAGACCAAC	CGACATCAAAGGTCCCTGGACCAAA	CGACATCAAAGGTCCCTGGACCAAA
Gata1	CCTGTGCAATGCCTGTGGCT	TGCCTGCCCGTTTGCTGACAA	GTATCACAAGATGAATGGTCAGAACC	CATTCGCTTCTTGGGCCGGATG
Fbn1	ACGTGGCGGGGAATGTACAAACA	CGTCAGAGCTGTGTAGCAGTAACCA	CGACTGTCAGCAGCTACTTCTGCAAAT	CGACTGTCAGCAGCTACTTCTGCAAAT
Col4a2	CGACCCTGGAAGCCCTGGATTTA	CGTTGTCTTCCCTTAAGTCCCAACA	CGATGGCAGGGATGCCTGGT	CGATGGCAGGGATGCCTGGT
Bmp4	CAGCCGAGCCAACACTGTGA	TGGGATGCTGCTGAGGTTGA	AGTTTCCATCACGAAGAACATCTGG	GAGGAAACGAAAAGCAGAGC
Acta2	AGGCACCACTGAACCCTAAG	CACAGCCTGAATAGCCACAT	CCAACCGGGAGAAAATGAC	ATGGCGGGGACATTGAAG
Cdh2	ATCAACAATGAGACTGGGGACATCA	CTTCCATGTCTGTGGCTTGAA	CACTGTGGCAGCTGGTCTGG	ATTAACGTATACTGTTGCACTTTCTCTCG
Serpine1	AAAACCCGGCGGCAGATCCA	CTTGTTCCACGGCCCCATGA	GATGCTATGGGATTCAAAGTCAA	CCTTGGAGAGCTGGCGGAGGGCATGA
Snai1	CGATCTGCACGACCTGTGGAAA	CGTGAGCGGTCAGCAAAAGCA	CGACTCTAGGCCCTGGCTGCTT	CGACTCTAGGCCCTGGCTGCTT
Snai2	CGAGCACATTCGAACCCACACA	CGTTGCAGTGAGGGCAAGAGAAA	CGATTGCCTTGTGTCTGCAAGA	CGATTGCCTTGTGTCTGCAAGA

Supplementary Figures Legends

Supplementary Figure S1. Analysis of the gene expression during in vitro EHT. A. FACS plot of day 1.5 BL-CFC culture showing VE-Cad and CD41 expression. Endothelial Cell (EC): VE-Cad^{+}CD41^{-}; Pre-Haematopoietic Progenitor Cell (Pre-HPC): VE-Cad^{+}CD41^{+}; Haematopoietic Progenitor Cell (HPC): VE-Cad^{-}CD41^{+}. **B.** Box plots comparing the expression of endothelial genes Cdh5, Kdr and Pecam1 between the 3 populations in A. **C.** Box plots comparing the expression of haematopoietic transcription factors Runx1, Myb and Gata1 between the 3 populations in A. The box plots were generated from 3 independent experiments. For each plot, the top and bottom box edges correspond to the first and third quartiles. The black line inside the box represents the median. The top and bottom whisker lines mark the maximum and minimum values of the data set, respectively. The corresponding p-values were calculated with Student's t-test (Supplementary Table S1).

Supplementary Figure S2. Analysis of the secretome LC-MS/MS data. A. Proteins detected in the supernatant after LC-MS/MS for the 3 biological replicates. **B.** Scatterplot showing pairwise comparison between the 3 replicates. The red dots correspond to the proteins whose expression is significantly different between TGFβ2 and control conditions (p.adj-value<0.05).

Supplementary Figure S3. Gene ontology (GO) analysis of the genes coding for the 33 secreted proteins detected in all 3 biological replicates. For each GO term, the corresponding genes are shown in red in a String representation of a network involving the secreted proteins (see Fig. 2). Different line colours represent the types of evidence for the association between proteins.

Supplementary Figure S4. Experimental layout for the experiments described in Figures 3 and 4.

Supplementary Figure S5. Inhibition of the TGFβ pathway with a dox inducible GFP-Smad7 ESC line. A. Scheme describing the dox inducible GFP-Smad7 ES cell line. **B.** Histogram of FACS data showing the expression of GFP-Smad7 upon doxycycline treatment. **C.** Bar graph showing the average frequency of VE-Cad^{+}CD41^{-} and VE-Cad^{-}CD41^{+} cell populations following 24h of treatment with SB431542 and dox (induction of Smad7 expression) in the first day of BL-CFC culture. The average was calculated from 3 independent experiments. The p-values were calculated with Student's t-test (2 tails, type 3). VE-Cad^{+}CD41^{-} population: * "Control" versus "+Dox" p-value = 0.013 (n=3); * "Control" versus "SB431542" p-value = 0.024 (n=3); VE-Cad^{-}CD41^{+} population: * "Control" versus "+Dox" p-value = 0.02 (n=3). **C.** Quantification of the number of round cells generated during time-lapse imaging in the 2 indicated conditions. Each value represents the mean of the number of round cells for 9 areas of the same well.

Supplementary Figure S6. Heatmap of gene expression from microarray analysis of Figure 4. A. Annotation of the microarray samples used for the study. **B.** Heatmap of gene expression for the top 250 genes differentially expressed following TGFβ signaling activation and inhibition.

Supplementary Figure S7. Analysis of the gene expression during in vivo EHT. A. FACS plots of E11 Aorta Gonad Mesonephros (AGM) region showing VE-Cad, Kit and CD41 expression. Endothelial Cell (EC): VE-Cad^{+}CD41^{-}; Pre-Haematopoietic Progenitor Cell (Pre-HPC): VE-Cad^{+}CD41^{+}; Haematopoietic Progenitor Cell (HPC): VE-Cad^{-}CD41^{+}Kit^{+}. **B.** Box plots comparing the expression of endothelial genes between the 3 populations in A. **C.** Box plots comparing the expression of haematopoietic genes between the 3 populations in A. The box plots were generated from 4 independent experiments. For each plot, the top and bottom box edges correspond to the first and third quartiles. The black line inside the box represents the median. The top and bottom whisker lines mark the maximum and minimum values of the data set, respectively. The corresponding p-values were calculated with Student's t-test (Supplementary Table S8).

Supplementary Figure S8. q-RT-PCR validation of genes found differentially expressed after activation of the TGFβ pathway. A. Box plots comparing the expression of vasculature related genes between the Activator, Control and Inhibitor conditions in EC, Pre-HPC and HPC populations. **B.** Box plots comparing the expression of EMT genes between the Activator, Control and Inhibitor conditions in EC, Pre-HPC and HPC populations. The box plots were generated from 3 independent experiments. For each plot, the top and bottom box edges correspond to the first and third quartiles. The black line inside the box represents the median. The top and bottom whisker lines mark the maximum and minimum values of the data set, respectively. The corresponding p-values were calculated with Student's t-test (Supplementary Table S1).

Supplementary Figure S9. Characterisation of the dox inducible Sox17-mCherry ES cell line. A. Scheme describing the dox inducible Sox17-mCherry ES cell line. **B.** Histogram of FACS data showing the expression of Sox17-mCherry upon doxycycline treatment. **C.** Quantification of the number of round cells generated during time-lapse imaging in the 2 indicated conditions. Each value represents the mean of the number of round cells for 7 areas of the same well. **D.** Box plots comparing the expression of haematopoietic genes between the 2 conditions. The box plots were generated from 3 independent experiments. For each plot, the top and bottom box edges correspond to the first and third quartiles. The black line inside the box represents the median. The top and bottom whisker lines mark the maximum and minimum values of the data set, respectively. The corresponding p-values were calculated with Student's t-test (Supplementary Table S9).

Supplementary Table Legends

Supplementary Table S1. P-values from T-test for the q-RT-PCR results shown in Figure 1, Supplementary Figure S1 and Supplementary Figure S8. The p-values were calculated with a T-test (2 tails, type 3) on 3 independent biological replicates. The p-values below 0.05 are marked in red.

Supplementary Table S2. Results of the analysis of the secretome LC-MS/MS data. See Excel file “Supplementary Table S2.xlsx”. This file contains 3 spreadsheets. The first one contains all secretome data. The second contains the list of proteins found with a fold discovery rate (FDR) of 5% in at least 2 replicates. The third contains the list of proteins found with a fold discovery rate (FDR) of 5% in 3 replicates.

Supplementary Table S3. Log_2 fold change TGFβ2/control for the 33 proteins detected in the all 3 biological replicates.

Supplementary Table S4. Results of the microarrays data from Figure 4. See Excel file “Supplementary Table S4.xlsx”. This file contains 2 spreadsheets. The first one contains the results of the gene expression comparison between activator (TGFβ2) and control samples. The second one contains the results of the gene expression comparison between inhibitor (SB431542) and control samples.

Supplementary Table S5. Gene ontology analysis of the gene up-regulated and down-regulated after TGFβ2 treatment in the microarrays data from Figure 4. See Excel file “Supplementary Table S5.xlsx”. This file contains 2 spreadsheets. The first one contains DAVID gene ontology analysis of the genes up-regulated between activator (TGFβ2) and control samples. The second one contains DAVID gene ontology analysis of the genes down-regulated between activator (TGFβ2) and control samples.

Supplementary Table S6. Gene ontology analysis of the gene down-regulated after SB431542 treatment in the microarrays data from Figure 4. See Excel file “Supplementary Table S6.xlsx”. This file contains the results of the DAVID gene ontology analysis of the genes down-regulated between inhibitor (SB431542) and control samples.

Supplementary Table S7. P-values from T-test for the q-RT-PCR results shown in Figure 5. The p-values were calculated with a T-test (2 tails, type 3) on 4 independent biological replicates. The ones below 0.05 are marked in red.

Supplementary Table S8. P-values for the q-RT-PCR results shown in Figure 6 and Supplementary Figure S7. The p-values were calculated with a T-test (2 tails, type 3) on 4 independent biological replicates. The ones below 0.05 are marked in red.

Supplementary Table S9. P-values for the q-RT-PCR results shown in Figure 7 and Supplementary Figure S9. The p-values were calculated with a T-test (2 tails, type 3) on 3 independent biological replicates. The ones below 0.05 are marked in red.

Supplementary Table S10. List of primers used for q-RT-PCR done in Figure 5, Figure 7 and Supplementary Figure S9.

Supplementary Table S11. List of primers used for q-RT-PCR done in Figure 1, Figure 6, Supplementary Figure S7 and Supplementary Figure S8.

Supplementary Video Legends

Supplementary video S1. Time-lapse microscopy of EHT in normal medium. This movie shows Pre-HPC (VE-Cad^{+}CD41^{+}) going through EHT in haemogenic endothelium medium. Images were taken every 15 minutes for 64 hours. The video has 10 frames per second (Avi file, 4.5 MB).

Supplementary video S2. Time-lapse microscopy of EHT in medium supplemented with DMSO. This movie shows Pre-HPC (VE-Cad^{+}CD41^{+}) going through EHT in haemogenic endothelium medium with DMSO. Images were taken every 15 minutes for 64 hours. The video has 10 frames per second (Avi file, 7.4 MB).

Supplementary video S3. Time-lapse microscopy of EHT in medium supplemented with TGFβ2. This movie shows Pre-HPC (VE-Cad^{+}CD41^{+}) going through EHT in haemogenic endothelium medium with TGFβ2. Images were taken every 15 minutes for 64 hours. The video has 10 frames per second (Avi file, 8.2 MB).

Supplementary video S4. Time-lapse microscopy of EHT in medium supplemented with SB432542. This movie shows Pre-HPC (VE-Cad^{+}CD41^{+}) going through EHT in haemogenic endothelium medium with SB432542. Images were taken every 15 minutes for 64 hours. The video has 10 frames per second (Avi file, 10.2 MB).

Supplementary video S5. Time-lapse microscopy of EHT in medium without dox. This movie shows Pre-HPC (VE-Cad^{+}CD41^{+}) from iSox17 ESC going through EHT in haemogenic endothelium medium without dox. Images were taken every 15 minutes for 48 hours. The video has 10 frames per second (Avi file, 9.2 MB).

Supplementary video S6. Time-lapse microscopy of EHT in medium with dox. This movie shows Pre-HPC (VE-Cad^{+}CD41^{+}) from iSox17 ESC going through EHT in haemogenic endothelium medium with dox. Images were taken every 15 minutes for 48 hours. The video has 10 frames per second (Avi file, 6.5 MB).
